# Taurine, homotaurine, GABA and hydrophobic amino acids content influences “in vitro” antioxidant and SIRT1 modulation activities of enzymatic protein hydrolysates from algae

**DOI:** 10.1038/s41598-022-25130-4

**Published:** 2022-12-02

**Authors:** Carlos Terriente-Palacios, Susana Rubiño, Maria Hortós, César Peteiro, Massimo Castellari

**Affiliations:** 1 IRTA, Food Safety and Functionality Programe, Finca Camps I Armet s/n, Monells, 17121 Girona, Spain; 2 Escuela Internacional de Doctorado de la Universidad Nacional de Educación a Distancia (EIDUNED), Calle Bravo Murillo 38, 28015 Madrid, Spain; 3 Spanish Institute of Oceanography of the Spanish National Research Council (IEO, CSIC), Oceanographic Center of Santander, Marine Culture Units “El Bocal”, Seaweeds Center, Barrio Corbanera s/n., Monte, 39012 Santander, Spain

**Keywords:** Natural products, Enzymes

## Abstract

Prevention and control of diseases and delaying the signs of ageing are nowadays one of the major goals of biomedicine. Sirtuins, a family of NAD^+^ dependent deacylase enzymes, could be pivotal targets of novel preventive and therapeutic strategies to achieve such aims. SIRT1 activating and inhibiting compounds, such as polyphenols and bioactive peptides, have been proposed to be involved in the development of many human diseases. The objective of this work was to assess and compare the antioxidant and SIRT1 modulation activities of enzymatic protein hydrolysates (EPHs) from a wide number of algae species (24 commercial samples and 12 samples harvested off the Atlantic coast of northern Spain). High antioxidant activities were observed in EPHs from red and green seaweed species. Moreover, 19 samples exhibited SIRT1 activation, while EPHs from the 16 samples were SIRT1 inhibitors. Pearson's correlation test and Principal Component Analysis revealed significant correlations between (1) total peptide and hydrophobic amino acid content in EPHs and their antioxidant activities, and (2) concentrations of taurine, homotaurine, and amino acid gamma aminobutyric acid in EPHs and their SIRT1 modulation activity.

## Introduction

The ocean covers more than 70% of the earth's surface, and therefore marine biodiversity is an essential part of the global system since a large part of human food consumption is derived from marine resources, and global food demands are continually rising. Due to the complex habitats in which they live, marine organisms can produce a wide variety of secondary metabolites that cannot be found elsewhere. Hence, sponges, algae, and bryozoans, among many other marine organisms, are important sources of bioactive compounds with interesting properties like antioxidant, antimicrobial, anticancer, antihypertensive, and anti-inflammatory activities^1,2^.

Protein hydrolysates from macro-and microalgae have been reported by various authors as possible therapeutic agents due to their antimicrobial and antiviral^3,4^, anticancer^5^, antioxidant^6–9^, and immunoregulatory functions^10^. Additionally, a close relationship has been observed between the antioxidant capacity of the protein hydrolysates and their hydrophobic and/or aromatic amino acid content^11–14^. Heo et al.^7^ observed pronounced anti-oxidative effects in water-soluble, enzymatic extracts of seven species of marine edible brown seaweed from South Korea's coasts. In this study, protease extracts of *Ecklonia cava* scavenged DPPH free radicals more effectively than other algal extracts. Other authors^8,15^ found proteases more effective than carbohydrases in enhancing the recovery of hydrophilic antioxidant compounds from the seaweeds *Palmaria palmata* and *Undaria pinnatifida*, providing extracts with the greatest scavenging activity against DPPH and peroxyl radicals. Besides, Hu et al.^9^, observed that large hydrolysis times with a neutral protease enhanced the antioxidant activities of protein extracts from the microalgae *Schizochytrium limacinum*.

Sirtuins are a class of signaling NAD+ dependent proteins that may remove acetyl groups from histones^16^. The first identified sirtuin was the Silent Information Regulator 2 (SIR2/SIRT1), discovered in *Saccharomyces cerevisiae*, which was associated with the lifespan extension observed under dietary restriction in yeast^17^. Mice and humans express seven sirtuins (SIRT1-7) which possess either mono-ADP ribosyl-transferase or deacetylase activity.

It seems that SIRT1 and SIRT6 have a synergistic association in the spatiotemporal regulation of the DNA damage response and DNA repair mechanisms^18^. Several studies have shown that sirtuins can modulate cell response to oxidative or genotoxic stresses and play an important role in regulating sphingolipid metabolism^19,20^, as well as protecting against oxidative stress-mediated pathological processes such as ischemia–reperfusion, cardiac damage, arterial wall remodeling, inflammation, vascular aging, atherosclerosis, and brain senescence^21,22^. In addition, sirtuins are important for neural connectivity and synaptic plasticity; during aging, a deficit of SIRT1 activity has been directly associated with defined neurophysiological and neuropathological mechanisms of cognitive decline and metabolic dysfunction, and it has been stated that SIRT1 overexpression in certain neurons in the brain increases lifespan^23^.

In recent years, there has been a growing interest in the discovery of novel natural or synthetic SIRT1 modulators. Hence, SIRT1 activators (Liriopesides B, Schisandrin A) and inhibitors (3,5-dicaffeoylquinic acid, esculetin) have been previously identified in traditional medicine products^24^.

In streptozotocin-induced diabetic rats treated with horsetail extracts (*Equisetum arvense* L.), Hegedus et al.^25^ discovered increased SIRT1 levels in cardiomyocytes as well as anti-diabetic and cardioprotective effects. Huang et al.^26^ identified Ginsenoside Rc, an active ingredient of Panax ginseng, as a potent SIRT1 activator that promotes energy metabolism to improve cardio- and neuroprotective functions. However, no information has been found regarding seaweeds or microalgae protein hydrolysates extracts with sirtuin activation or inhibition capabilities.

Due to these multiple effects on metabolic functions, the identification of suitable sirtuin activating compounds (STACs) is a research field of great interest. Several classes of plant and seaweed polyphenols may increase SIRT1 activity^27,28^. Resveratrol (3, 5, 4′-trihydroxystilbene), a polyphenol found in grapes and red wines, is one of the most effective SIRT1 activators^29^. Taurine (2-aminoethanesulfonic acid, Tau), Homotaurine (Htau), and the amino acid gamma aminobutyric acid (GABA), have been recently proposed as natural SIRT1 activators with possible therapeutic benefits for metabolic, age-related, and neurological disorders^30–32^. Besides, phlorotannin-rich natural extract from the brown seaweed *Ascophyllun nodosum* and polyphenol extract from *Ecklonia cava* have been studied for their ability to increase SIRT1 activity^33,34^. Other studies, on the other hand, have shown that inhibiting or decreasing SIRT1 can inhibit cancer cell proliferation^35,36^. Furthermore, some studies have found that peptides and protein fractions can modulate sirtuin activities, which can slow the aging process^37–39^.

Tau, a β-amino acid found in very high concentration in most cells, has been pointed out as a promising new therapeutic agent in the treatment of several diseases affecting the muscles, the central nervous and the cardiovascular systems as well as with potent antitumor activities to control, e.g., hepatocellular carcinoma^40–42^. In addition, Tau was also demonstrated to improve the antioxidant defence networks by scavenging free radicals, maintaining the integrity of the electron-transport chain of mitochondria, inhibiting the activities of ROS-producing enzymes, etc.^43^. Htau, an aminesulfonate compound and an analog of GABA naturally found in several algae species, has been previously described as a potent neuroprotector and as a possible therapeutic agent for Alzheimer’s disease, Parkinson’s disease, and Mild cognitive impairment^44^. Finally, GABA is a potent inhibitory neurotransmitter in the central nervous system in animals and a powerful protector against stress in plants and microorganisms. In mammals, interesting health implications have been described such as epilepsy, depression and cancer, and in neural diseases such as schizophrenia, Parkinson’s disease, Alzheimer’s disease, and Huntington´s disease^45^.

The aim of this work was to assess and compare the antioxidant and SIRT1 modulation activities of enzymatic protein hydrolysates from 36 samples of different algae species, being for most of these algal species the first time that their protein hydrolysates have been assessed for these activities. Additionally, possible correlations between Taurine, homotaurine and GABA levels in the EPHs, with their antioxidant and / or SIRT1 modulation capabilities have been evaluated.

## Results and discussion

### Composition of algae protein hydrolysates

#### Protein content

The protein content in algae samples and in their corresponding protein isolates is summarized in Table 1. The green algae group, especially the microalgae *Aphanizomenon flos-aquae*, *Arthrospira platensis*, *Chlorella vulgaris*, and *Auxenochlorella pyrenoidosa,* showed the highest total protein concentration in both dried samples and in their protein hydrolysates (EPHs). These results agree with previous studies^46^, which indicate microalgae represent a good source of high-quality protein for human food consumption.

**Table 1:** Protein content (in the algae samples and in their protein isolates) and protein recovery (mean of n = 3 independent determinations ± SD).

	Protein (g/100 g d.w.)		Protein recovery %
	Algae	Protein isolate	
**Red algae**			
*Porphyra *sp.	31.0 ± 1.25	15.6 ± 0.41	50.3 ± 0.47
*Gigartina pistillata*	28.1 ± 2.65	15.7 ± 0.63	55.9 ± 0.65
*Chondrus crispus*	12.8 ± 1.05	6.19 ± 1.25	48.4 ± 0.98
*Mastocarpus stellatus*	11.0 ± 1.33	5.10 ± 1.27	46.4 ± 1.08
*Palmaria palmata*	17.8 ± 1.21	10.4 ± 0.47	58.4 ± 1.65
*Gelidium corneum*	9.42 ± 0.58	5.41 ± 0.69	57.4 ± 1.33
*Plocammium cartilagineum*	10.2 ± 0.66	6.41 ± 0.98	62.8 ± 2.22
*Centroceras clavulatum*	11.1 ± 1.65	7.03 ± 1.65	63.3 ± 2.25
*Halopithys incurva*	13.5 ± 1.87	7.41 ± 1.74	54.9 ± 2.39
*Median*	**12.8**^**b**^	**7.03**^**b**^	**55.9**^**a**^
**Green algae**			
*Aphanizomenon flos-aquae*	36.4 ± 1.55	21.2 ± 1.41	58.2 ± 1.65
*Caulerpa lentillifera*	13.2 ± 1.63	7.74 ± 1.66	58.6 ± 1.54
*Codium *sp.	10.8 ± 1.74	5.87 ± 1.87	54.3 ± 1.74
*Dunaliella salina*	18.1 ± 1.04	11.8 ± 2.08	65.0 ± 2.55
*Arthrospira platensis*	33.0 ± 1.10	22.7 ± 2.56	68.8 ± 2.14
*Chlorella vulgaris*	50.3 ± 2.02	30.5 ± 2.44	60.6 ± 2.05
*Tetraselmis chui*	14.2 ± 2.23	9.60 ± 1.65	67.6 ± 1.32
*Auxenochlorella pyrenoidosa*	36.0 ± 2.85	23.9 ± 2.39	66.4 ± 1.33
*Ulva lactuca*	26.3 ± 1.95	15.4 ± 2.77	58.5 ± 1.22
*Ulva intestinalis*	9.28 ± 1.33	6.35 ± 2.41	68.5 ± 2.85
*Codium decorticatum*	9.35 ± 1.47	5.41 ± 1.65	57.9 ± 1.96
*Median*	**18.1**^**a**^	**11.8**^**a**^	**65.0**^**a**^
**Brown algae**			
*Ascophyllum nodosum*	8.30 ± 1.54	4.13 ± 1.65	49.7 ± 1.36
*Sargassum fusiforme*	21.2 ± 2.22	7.83 ± 2.36	36.9 ± 1.52
*Eisenia bicyclis*	12.0 ± 2.47	8.11 ± 1.41	67.6 ± 3.25
*Laminaria ochroleuca*	8.41 ± 2.69	4.57 ± 1.44	54.3 ± 1.69
*Himanthalia elongata*	10.2 ± 1.36	3.20 ± 2.41	31.4 ± 2.66
*Undaria pinnatifida*	12.4 ± 1.55	7.83 ± 3.33	63.1 ± 2.45
*Odontella aurita*	14.3 ± 1.87	6.58 ± 3.08	46.0 ± 2.74
*Fucus vesiculosus*	11.8 ± 2.52	6.95 ± 2.52	58.9 ± 2.52
*Bifurcaria bifurcata*	10.9 ± 2.05	6.72 ± 3.10	61.6 ± 2.41
*Fucus guiryi*	13.5 ± 2.07	9.11 ± 2.15	67.5 ± 1.65
*Pelvetia canaliculata*	7.56 ± 1.35	4.92 ± 3.25	65.1 ± 1.66
*Halopteris scoparia*	8.65 ± 1.66	5.15 ± 2.05	59.5 ± 2.65
*Gongolaria baccata*	12.7 ± 1.65	6.45 ± 2.55	50.8 ± 2.85
*Cladostephus spongiosus*	7.44 ± 2.36	4.78 ± 2.98	64.2 ± 3.05
*Ericaria selaginoides*	13.2 ± 1.23	8.49 ± 2.12	64.3 ± 1.05
*Nannochloropsis *sp.	37.0 ± 1.05	23.8 ± 1.52	64.3 ± 2.55
*Median*	**11.9**^**b**^	**6.65**^**b**^	**60.6**^**a**^

Significant values are in bold.

Different small letters in the same column indicate significant differences between medians of the algae groups (*p* < 0.05).

The recovery of the protein fraction in algae hydrolysates was comprised of between 31.4% and 68.8%, with no significant differences between algae groups. These results are in line with Hu et al.^9^, who obtained enzymatic protein hydrolysates from microalgae species with protein recoveries ranging between 40 and 60%.

#### Peptide concentration

EPHs of red and green species showed the highest peptide content (Table 2); among the 36 samples, *Aphanizomenon flos-aquae*, *Chlorella vulgaris*, and *Ulva intestinalis* showed peptide concentrations higher than 2.8 mg glutathione equivalent/mL.

**Table 2:** Peptide, sum of hydrophobic amino acids (SHA), Taurine (Tau), Homotaurine (Htau), and Gamma-aminobutyric acid (GABA) content in enzymatic protein hydrolysates (EPH) from the 36 algae species studied (mean of n = 3 independent determinations ± SD)

	Peptide	SHA	Tau	Htau	GABA
	mg Glu eq/mL		g/100 g d.w		
**Red algae**					
*Porphyra *sp.	2.32 ± 0.14	21.5 ± 2.13	0.35 ± 0.02	0.06 ± 0.01	1.25 ± 0.08
*Gigartina pistillata*	2.05 ± 0.13	20.7 ± 3.65	0.38 ± 0.02	0.05 ± 0.00	1.14 ± 0.01
*Chondrus crispus*	2.11 ± 0.09	21.8 ± 1.52	0.25 ± 0.01	0.04 ± 0.00	1.05 ± 0.11
*Mastocarpus stellatus*	1.98 ± 0.21	20.0 ± 2.54	0.14 ± 0.01	0.04 ± 0.01	0.88 ± 2.74
*Palmaria palmata*	1.98 ± 0.21	28.4 ± 2.74	0.06 ± 0.01	0.02 ± 0.01	0.35 ± 0.02
*Gelidium corneum*	2.58 ± 0.19	48.2 ± 2.96	0.03 ± 0.01	0.01 ± 0.01	0.39 ± 0.01
*Plocammium Cartilagineum*	2.64 ± 0.11	33.0 ± 3.05	0.04 ± 0.01	0.02 ± 0.01	0.29 ± 0.06
*Centroceras clavulatum*	2.14 ± 0.10	44.9 ± 1.74	0.02 ± 0.04	0.00 ± 0.01	0.19 ± 0.05
*Halopithys incurva*	2.81 ± 0.04	47.0 ± 2.65	0.04 ± 0.01	0.02 ± 0.01	0.37 ± 0.03
*Median*	**2.14**^**a**^	**28.4**^**a**^	**0.06**^**b**^	**0.02**^**a**^	**0.39**^**b**^
**Green algae**					
*Aphanizomenon flos-aquae*	2.83 ± 0.14	26.7 ± 2.52	0.24 ± 0.01	0.04 ± 0.00	1.05 ± 0.08
*Caulerpa lentillifera*	2.28 ± 0.10	28.7 ± 2.74	0.39 ± 0.02	0.06 ± 0.00	1.28 ± 0.09
*Codium *sp.	1.84 ± 0.05	9.90 ± 2.04	0.38 ± 0.01	0.06 ± 0.00	1.27 ± 0.05
*Dunaliella salina*	2.13 ± 0.06	21.5 ± 3.12	0.35 ± 0.00	0.04 ± 0.00	0.91 ± 0 .03
*Arthrospira platensis*	2.60 ± 0.04	26.6 ± 3.63	0.35 ± 0.03	0.05 ± 0.00	0.83 ± 0.05
*Chlorella vulgaris*	2.87 ± 0.15	26.5 ± 3.55	0.31 ± 0.00	0.04 ± 0.00	0.94 ± 1.05
*Tetraselmis chui*	1.62 ± 0.17	13.1 ± 2.55	0.25 ± 0.05	0.05 ± 0.00	0.63 ± 0.20
*Auxenochlorella pyrenoidosa*	2.43 ± 0.09	26.8 ± 2.74	0.27 ± 0.00	0.04 ± 0.00	0.72 ± 0.92
*Ulva lactuca*	2.38 ± 0.17	14.9 ± 1.85	0.03 ± 0.00	0.02 ± 0.00	0.37 ± 0.03
*Ulva intestinalis*	2.81 ± 0.13	45.5 ± 1.96	0.26 ± 0.01	0.05 ± 0.01	0.82 ± 0.01
*Codium decorticatum*	1.67 ± 0.15	16.9 ± 1.47	0.00 ± 0.00	0.01 ± 0.00	0.18 ± 001
*Median*	**2.38**^**a**^	**21.5**^**a**^	**0.27**^**a**^	**0.04**^**a**^	**0.88**^**a**^
**Brown algae**					
*Ascophyllum nodosum*	1.51 ± 0.04	4.70 ± 0.05	0.19 ± 0.00	0.05 ± 0.00	0.68 ± 0.02
*Sargassum fusiforme*	1.70 ± 0.14	5.00 ± 0.55	0.25 ± 0.00	0.04 ± 0.00	0.72 ± 0.01
*Eisenia bicyclis*	1.86 ± 0.16	6.60 ± 0.41	0.23 ± 0.00	0.03 ± 0.00	0.74 ± 0.00
*Laminaria ochroleuca*	2.19 ± 0.18	27.2 ± 3.52	0.25 ± 0.01	0.05 ± 0.00	0.85 ± 0.01
*Himanthalia elongata*	1.69 ± 0.25	9.00 ± 1.55	0.23 ± 0.01	0.05 ± 0.00	0.65 ± 0.06
*Undaria pinnatifida*	1.73 ± 0.16	14.4 ± 1.47	0.19 ± 0.05	0.04 ± 0.00	0.64 ± 0.08
*Odontella aurita*	1.80 ± 0.08	27.2 ± 1.98	0.19 ± 0.01	0.04 ± 0.00	1.12 ± 0.13
*Fucus vesiculosus*	1.83 ± 0.27	18.6 ± 1.85	0.21 ± 0.01	0.03 ± 0.00	0.51 ± 0.03
*Bifurcaria bifurcata*	2.12 ± 0.20	31.3 ± 3.74	0.00 ± 0.00	0.01 ± 0.00	0.00 ± 0.00
*Fucus guiryi*	1.62 ± 0.05	4.60 ± 3.14	0.12 ± 0.00	0.01 ± 0.00	0.27 ± 0.02
*Pelvetia canaliculata*	1.72 ± 0.14	5.50 ± 3.85	0.07 ± 0.00	0.02 ± 0.00	0.09 ± 0.01
*Halopteris scoparia*	1.82 ± 0.10	4.60 ± 2.52	0.04 ± 0.00	0.01 ± 0.00	0.25 ± 0.12
*Gongolaria baccata*	1.88 ± 0.16	4.61 ± 2.15	0.06 ± 0.00	0.02 ± 0.00	0.11 ± 0.10
*Cladostephus spongiosus*	2.01 ± 0.18	9.81 ± 2.19	0.05 ± 0.00	0.01 ± 0.00	0.11 ± 0.01
*Ericaria selaginoides*	1.55 ± 0.21	5.42 ± 1.14	0.06 ± 0.01	0.02 ± 0.00	0.39 ± 0.01
*Nannochloropsis *sp.	2.22 ± 0.27	27.1 ± 1.11	0.15 ± 0.06	0.03 ± 0.00	0.49 ± 0.05
*Median*	**1.83**^**b**^	**5.50**^**b**^	**0.17**^**a**^	**0.03**^**a**^	**0.50**^**b**^

Significant values are in bold.

Different small letters in the same column indicate significant differences between medians of the algae groups (*p* < 0.05).

#### Amino acid profiles

The analysis of the amino acid profiles showed that red and green algae EPHs had the highest content in hydrophobic amino acids. Green algae EPHs contained significantly more GABA than the red and brown groups, while EPHs from both green and brown algae showed the highest contents in Tau (Table 2, and Table S1, *Supplementary material*). *Porphyra sp*., *Gigartina pistillata*, *Caulerpa lentillifera* and *Codium sp*. samples provided the EPHs with the highest amounts of Tau, Htau, and / or GABA. Considering the significance and health implications that these molecules have, it is worthwhile to quantify their content in protein hydrolysates. Liaset et al. and Lassoued et al., highlighted the high levels of Tau concentrations in fish protein hydrolysates^47,48^, while Evache et al.^49^ stated that seaweed protein hydrolysates could have significantly higher levels of Tau compared to other vegetal sources. However, in the scientific literature there is a substantial lack of information about the Tau, Htau and GABA content in enzymatic protein hydrolysates from algae.

#### Antioxidant activities

##### TEAC and DPPH assays

Strong antioxidant (TEAC) and antiradical (DPPH) activities (both higher than 70%) were observed in EPHs from 10 algae species (*Porphyra *sp., *Gigartina pistillata*, *Plocammium cartilagineum*, *Caulerpa lentillifera*, *Arthrospira plantensis*, *Chlorella vulgaris*, *Tetraselmis chui*, *Ulva lactuca*, *Ulva intestinalis,* and *Nannocloropsis *sp.).

EPHs from green and red species showed significantly higher median antioxidant (TEAC) and free radical scavenging (DPPH) activities than those of the brown species (Table 3). The highest TEAC and DPPH activities in the different groups were found in the EPHs of *Porphyra *sp., *Caulerpa lentillifera*, *Ulva* *intestinalis,* and *Bifurcaria bifurcata.*

**Table 3:** Antioxidant (TEAC, DPPH) activities in enzymatic protein hydrolysates (EPHs) from the algae samples (mean of n = 3 independent determinations ± SD).

	TEAC %	DPPH %
**Red algae**		
*Porphyra *sp.	71.2 ± 1.65	70.4 ± 0.15
*Gigartina pistillata*	54.1 ± 0.32	51.4 ± 0.36
*Chondrus crispus*	56.5 ± 0.11	50.2 ± 0.42
*Mastocarpus stellatus*	54.3 ± 0.12	53.2 ± 0.14
*Palmaria palmata*	54.6 ± 0.25	56.6 ± 1.65
*Gelidium corneum*	69.2 ± 0.36	67.4 ± 2.54
*Plocammium Cartilagineum*	68.8 ± 0.41	67.9 ± 2.14
*Centroceras clavulatum*	69.4 ± 0.09	68.8 ± 1.54
*Halopithys incurva*	68.5 ± 0.11	65.7 ± 0.85
*Median*	**68.5 **^**a**^	**65.7 **^**a**^
**Green algae**		
*Aphanizomenon flos-aquae*	65.4 ± 0.07	63.5 ± 2.10
*Caulerpa lentillifera*	72.1 ± 0.05	70.0 ± 2.52
*Codium *sp.	52.3 ± 0.15	55.8 ± 2.77
*Dunaliella salina*	60.2 ± 0.23	58.8 ± 1.41
*Arthrospira platensis*	70.2 ± 1.25	68.6 ± 3.65
*Chlorella vulgaris*	70.5 ± 2.36	68.8 ± 0.54
*Tetraselmis chui*	48.9 ± 1.87	50.1 ± 1.44
*Auxenoclorella pyrenoidosa*	65.5 ± 2.47	63.4 ± 2.58
*Ulva lactuca*	51.1 ± 2.87	52.3 ± 0.88
*Ulva intestinalis*	71.2 ± 0.08	73.6 ± 0.65
*Codium decorticatum*	60.5 ± 3.11	58.9 ± 0.96
*Median*	**60.5**^**a**^	**58.9**^**a**^
**Brown algae**		
*Ascophyllum nodosum*	40.7 ± 1.54	42.6 ± 2.22
*Sagassum fusiforme*	41.2 ± 3.36	45.2 ± 2.74
*Eisenia bicyclis*	47.8 ± 2.87	49.3 ± 0.98
*Laminaria ochroleuca*	59.1 ± 2.54	58.6 ± 1.11
*Himanthalia elongata*	47.7 ± 3.47	45.5 ± 2.41
*Undaria pinnatifida*	48.8 ± 1.74	50.1 ± 3.55
*Odonella aurita*	60.7 ± 1.23	58.5 ± 2.22
*Fucus vesiculosus*	54.3 ± 1.69	52.4 ± 2.64
*Bifurcaria bifurcata*	61.2 ± 3.44	60.3 ± 2.27
*Fucus guiryi*	46.4 ± 1.27	44.5 ± 1.55
*Pelvetia canaliculata*	49.7 ± 2.27	47.7 ± 1.48
*Halopteris scoparia*	39.5 ± 1.13	37.8 ± 2.28
*Gongolaria baccata*	45.5 ± 1.54	42.8 ± 3.24
*Cladostephus spongiosus*	50.2 ± 1.74	47.5 ± 1.05
*Ericaria selaginoides*	43.5 ± 2.28	41.9 ± 1.49
*Nanochloropsis *sp.	58.9 ± 2.14	60.1 ± 1.74
*Median*	**49.7**^**b**^	**47.5**^**b**^

Significant values are in bold.

Different small letters in the same column indicate significant differences between medians of the algae groups (*p* < 0.05).

These results are in good agreement with the studies of Je et al.^15^ and Norzagaray-Valenzuela et al.^50^, who observed high and comparable values of the TEAC and DPPH assays in algal protein hydrolysates from *Nannochloropsis sp*. and *Dunaliella tertiolecta* and relatively low free radical scavenging activities in Alcalase® extracts from *Undaria pinnatifida.* To the best of our knowledge, the antioxidant properties of EPHs from almost all the algae species included in the study (except *Undaria pinnatifida*^15^, *Ascophyllum nodosum*^27^, *Nannochloropsis *sp.^50^, and *Fucus vesiculosus*^51^) have never been evaluated.

Besides, our results are in line with the DPPH/TEAC activities observed in Alcalase® protein hydrolysates from other animal and plant sources, such insects^52^, scallop^53^, cuttlefish^54^, and black beans^55^, indicating that EPHs from algae show interesting antioxidative properties with potential applications in the food industry.

#### SIRT1 activity

**Figure 1:**
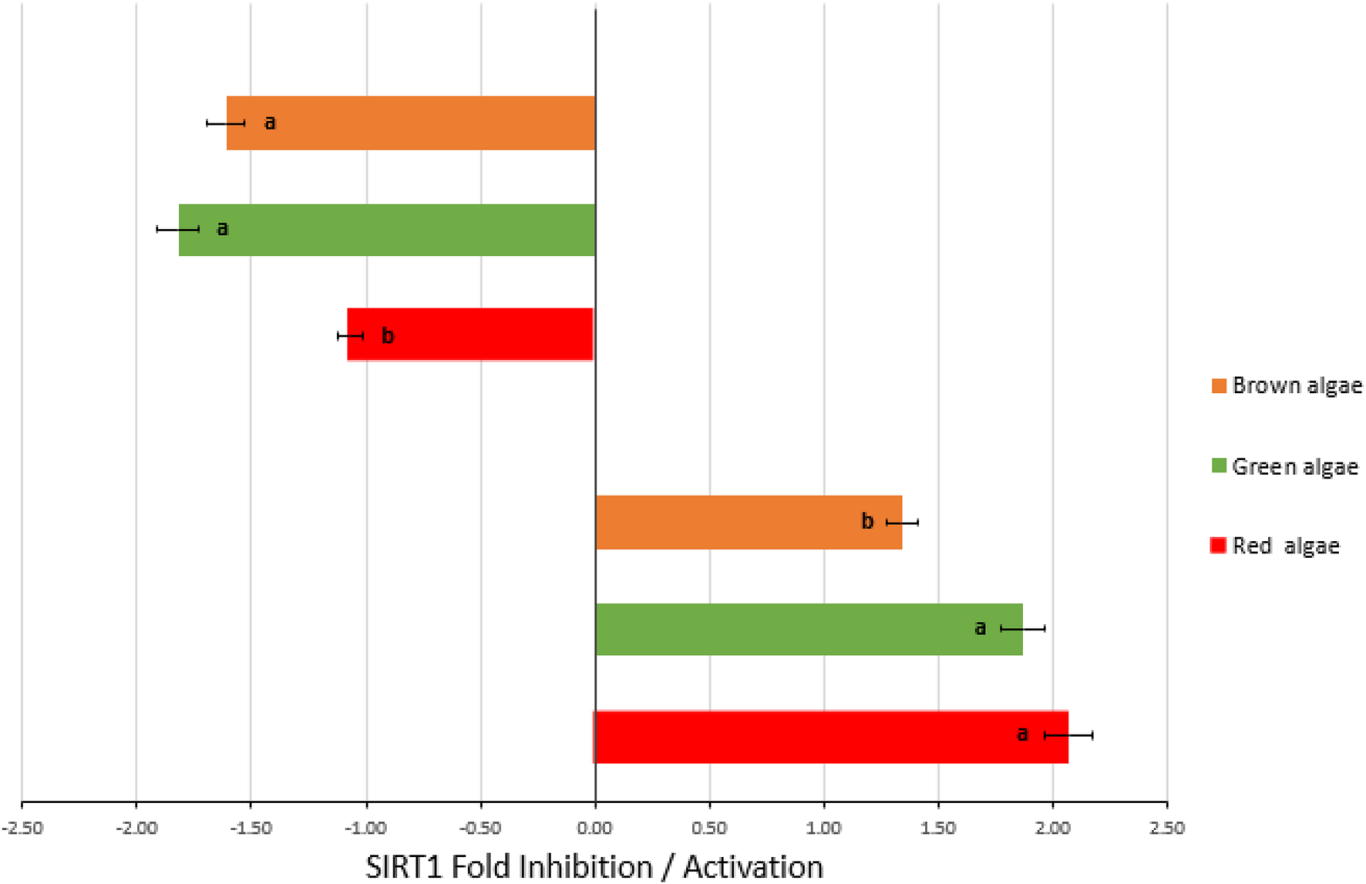
SIRT1 median fold activation / inhibition induced by EPHs from brown, red, and green algae. SIRT1 enzyme initial activity was used as a control. Different small letters indicate significant differences between algae groups (*p* < 0.05) with EPHs providing Fold inhibition. Different capital letters indicate significant differences between algae groups (*p* < 0.05) with EPHs providing Fold activation.

Figure 1 summarizes the results expressed as median fold activation or inhibition for the EPHs of each algae group. Significant differences (*p* < 0.05) were found between the algae groups, indicating that EPHs´ from the red and green algae groups were the most potent SIRT1 activators and / or inhibitors.

EPHs from *Porphyra *sp., *Caulerpa lentillifera*, *Codium sp*., and *Odontella aurita* were the most effective SIRT1 activating agents with values higher than 80%. Surprisingly, all the EPH from seaweed collected on the Atlantic coast of northern Spain but only four commercial species showed SIRT1 inhibition (Table S2, *Supplementary material*).

These results could be associated with the harvesting, drying, and storage conditions applied to "experimental" and commercial samples, which, even if no specific information was available on the labels of the commercial products, are likely to be quite different.

This hypothesis appears to be supported by studies indicating that different drying methods used on algal biomass have a significant impact on the nutritional and/or functional components of the final product (e.g., protein content and total antioxidant capacity)^56,57^.

In the case of samples inducing SIRT1 activation, all the three algae groups induced a median fold activation around 1.50 or higher (Fig. 1). Dutot et al.^33^ reported similar results working with an extract rich in phlorotannins from a commercial sample of *Ascophyllum nodosum*, while Fitton et al.^58^ found that a fucoidan-rich extract from *Undaria pinnatifida* increased SIRT1 expression of around 28.8%. SIRT1 fold activation results of around 1.30 have been previously described for natural compounds such as quercetin, ferulic acid, tyrosol, etc. at concentrations between 0.5 and 2.0 mg/mL^59^.

Algal EPHs that inhibited SIRT1 showed a median fold inhibition of around 1.65, corresponding to median % inhibition between 32 and 57% (Fig. 1 and Table S2, *Supplementary material*). Selisistat (EX – 527), a synthetic compound identified as the most potent SIRT1 inhibitor to date, can reach extremely high SIRT1 inhibition values up to 83.6% at 0.0125 mg/mL^60^. Besides, in a recent study^61^, two phenolic compounds, rhuschalcone I and rhuschalcone IV, previously isolated from the twigs and root bark of the medicinal plant *Rhus pyroides* Burch (Anacardiaceae), showed inhibitory effects against SIRT1 at 0.02 mg/ml (rhuschalcone I = 53.5%; rhuschalcone IV = 61.2%). In this study EPHs from algae reached similar effects, even if at higher concentrations (5 mg/mL).

Even if these results have been obtained “in vitro” and should be considered preliminary, they seem to indicate that EPHs from microalgae and seaweed included in this study had a substantial capacity to modulate SIRT1, especially considering they were not purified fractions.

### Correlation analysis and principal component analysis

**Figure 2:**
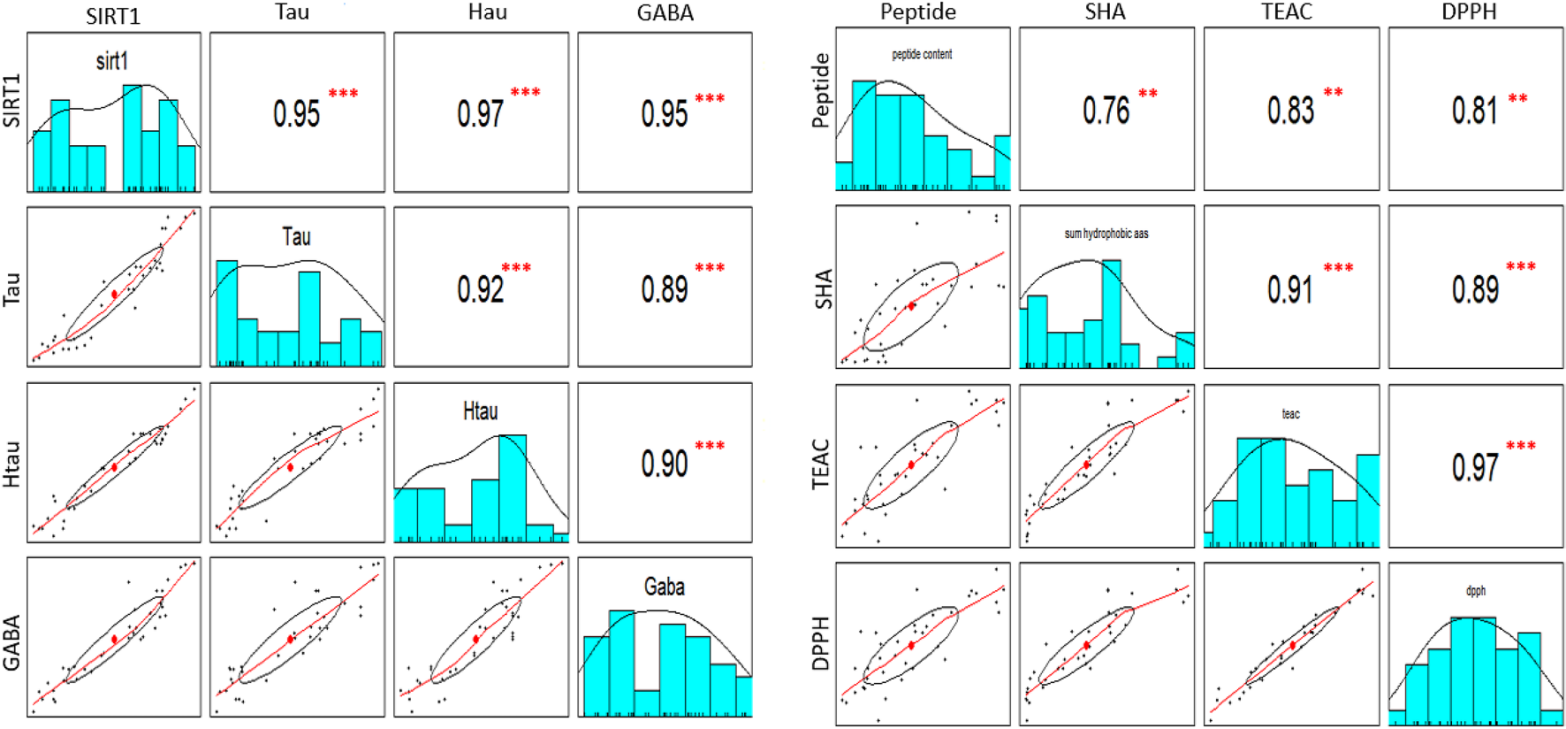
Pairwise scatter plot matrix, distribution, and Pearson correlation coefficients (R) for comparisons among specific amino acids, peptide content, antioxidant and SIRT1 activities in enzymatic protein hydrolysates from 36 algae species. On the bottom of the diagonal, the bivariate scatter plots with a fitted line, and on the top of the diagonal, the value of the correlation (R) plus the significance level as asterisks (**p < 0.01; ***p < 0.001). Peptide = Peptide concentration; SHA = Sum of hydrophobic amino acids; DPPH and ABTS = antioxidant activities; SIRT1 = EPH modulators of Sirtuin 1; GABA = Gamma amino butyric acid; Tau: taurine; Htau: Homotaurine.

Pearson’s Correlation Coefficients (PCCs, Fig. 2) showed highly significant correlations between peptide content with both TEAC and DPPH activities, in good agreement with previous findings reported in wheat germ and in the microalgae *Dunaliella salina*^62,63^. Additionally, a significant correlation between EPH hydrophobic amino acid content and TEAC and DPPH was observed in algae EPHs, as already described in other biological extracts; it was suggested that hydrophobic amino acids probably act as antioxidants by increasing the solubility of peptides in lipids and, therefore, facilitating their better interaction with free radicals that cause oxidative damage^64^.

Peptide and hydrophobic amino acids content showed a PCC of around 0.76, (*p* < 0.01), indicating that peptides in the protein hydrolysates contain a great proportion of these amino acids. Hence, our results are in line with other studies underlying hydrophobic amino acids originated by the hydrolysis of jumbo squid skin gelatin and giant squid muscle, or conger eel (*Conger myriaster*) contributed to the antioxidant activities of the peptide fractions^11–13^.

Results of TEAC and DPPH assays were highly correlated, in line with results observed in other studies carried out in methanolic extracts from several fruits, vegetables, and beverages^65^.

Significant, positive correlations were also observed between Tau, Htau and GABA concentrations in algae EPHs and SIRT1 modulation activity (Fig. 2).

This correlation has been previously observed in the study of KP et al.^30^, where Tau was established as a positive regulator of SIRT1 activity in hepatic cells “in vitro”. Our results also agreed with previous studies in human cultured cells where homotaurine and GABA significantly increased the expression of SIRT1^31,32^.

**Figure 3:**
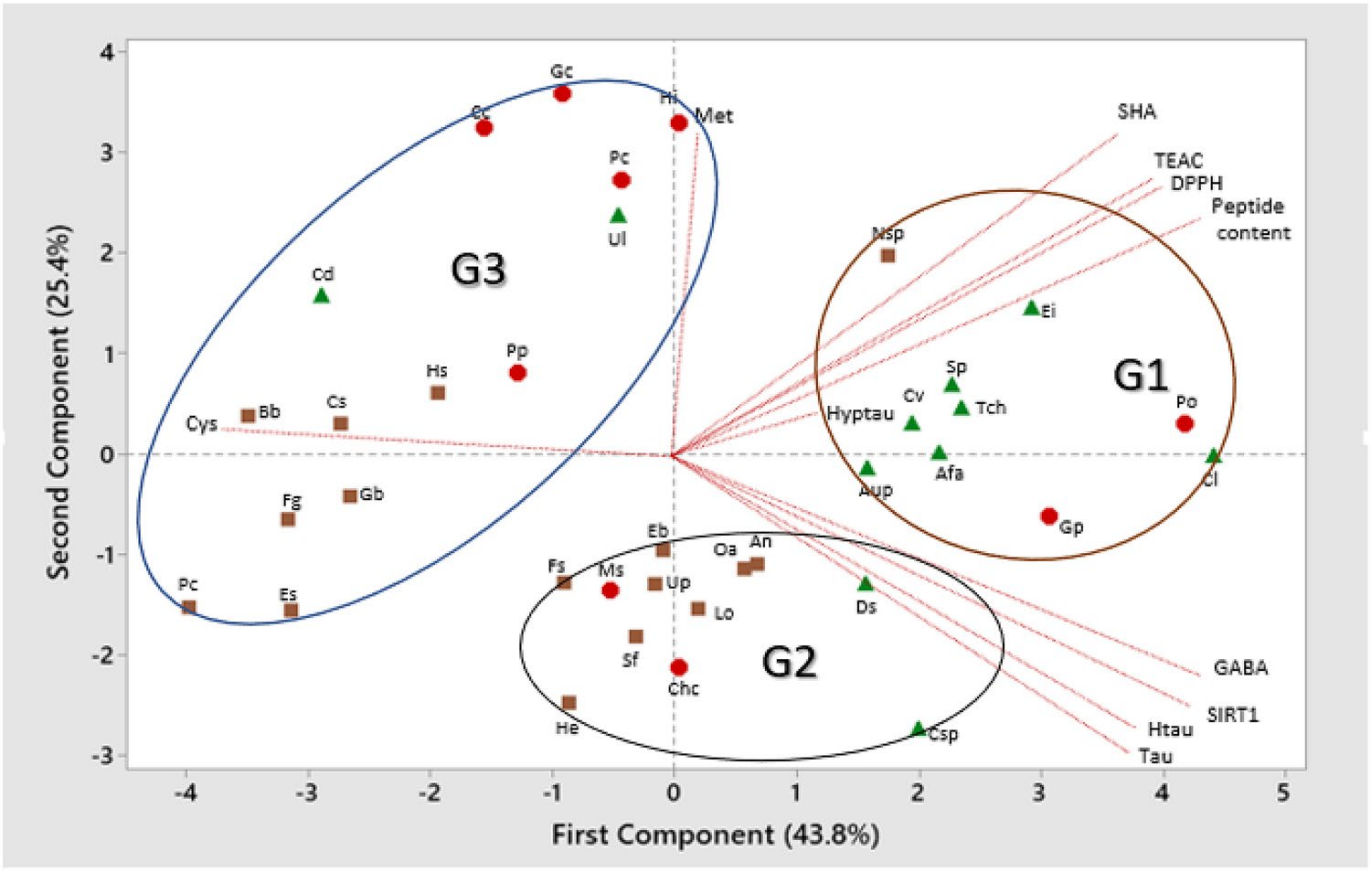
Principal Components Analysis of EPHs from 36 algae samples. Met = Methionine; SHaas = Sum of hydrophobic amino acids; DPPH and ABTS = antioxidant activities; SIRT1 = Sirtuin 1 inhibition/activation activity; GABA = Gamma amino butyric acid; Tau: taurine; Htau: Homotaurine; Hyptau: Hypotauine; Cys: Cysteine. Circles, squares, and triangles represent red, brown, and green algae species, respectively. Abbreviations of algae species as listed in Table 1.

A principal component analysis (PCA) was performed in order to detect structure in the relationship between the independent analytical variables and EPHs from the different algae samples. According to the Kaiser criterion the first two principal components (PCs) were the only significant ones and accounted for 43.8% and 25.4% of the total variance of the data, respectively. EPHs configuration and analytical attributes loadings (plotted as vectors) are presented for the first two PCs in Fig. 3. The combination of the two PCs discriminates three groups (G1–G3) of EPHs. G1 gathers EPHs with the highest content of hydrophobic amino acids, peptides and antioxidant activities. The EPHs grouped within G2 were characterized by high content of Tau, Htau, GABA, and SIRT1 activation capacity. On the other hand, EPHs grouped in G3 showed inhibition of the SIRT1 activity, the lowest antioxidants capacities and low contents of Tau, Htau, GABA and hydrophobic amino acids.

## Conclusions

Food ingredients and extracts from algae are an interesting field of research due to their richness in different classes of bioactive compounds. However, there is a substantial lack of knowledge regarding the capacity of algae EPHs to modulate sirtuins activities, which has been pointed out for their influence on cell aging and metabolism.

To the best of our knowledge, it is the first time that EPHs antioxidant and SIRT1 modulation activities have been evaluated in most of the algae species included in this study.

Our results indicated that EPHs from a broad range of algae species provided significant antioxidant and SIRT1 modulation activities. SIRT1 activation was significantly correlated to the content of Tau, Htau and GABA in EPHs, while antioxidant activities (TEAC and DPPH) were significantly correlated to the total hydrophobic amino acids in algal EPH.

The main conclusion of this study is that enzymatic hydrolysates from water-soluble proteins of different algae species are a potential source of ingredients with interesting functional properties that could find suitable applications in both the food and pharmaceutical industries. EPHs obtained from certain algae species (*Caulerpa lentillifera*, *Codium sp*.) showed strong SIRT1 activation capacity, which has been described as a protective mechanism against certain diseases such as metabolic syndrome, cardiovascular diseases, and neurodegeneration^21–23,26^. On the other hand, other EPHs (*Bifurcaria bifurcata*, *Codium decorticatum*) exhibited high SIRT1 inhibition values, and several studies showed that decreasing SIRT1 can inhibit cancer cell proliferation^35,36^.

Hence, further studies are needed in order to purify and further characterize target compounds in EPHs responsible for the observed antioxidant and SIRT1 modulation activities, investigate their effects "in vivo" as well as to clarify the influence of algae biomass storage and processing on the EPHs composition and their functional activities.

## Materials and methods

### Chemicals and reagents

All the chemicals used in the experiments were of analytical grade. Acetonitrile (ACN) and methanol (MeOH) were HPLC gradient-grade (Merck KGaA (Darmstadt, Germany). Per-chloric (60%) and hydrochloric acid (37%) were from J.T. Baker (NJ, United States). Sigma-Aldrich Chemie (Sant Quentin Fallavier, France) provided Alcalase 2.4 L FG (CAS no. 9014-01-1), ortho-phthaldialdehyde reagent (OPA), l-glutathione, borate buffer, formic acid (for LC-MS LiChropur™), ammonium acetate, ammonium formate, phenyl isothiocyanate (PITC), triethylamine (TEA), pure standards for 19 amino acids, taurine, hypotaurine and homotaurine, trolox (6-hydroxy-2,5,7,8-tetramethylchromane-2-carboxylic acid), 2,20-azinobis-(3-ethylbenzothiazoline-6-sulfonic acid) diammonium salt (ABTS), α-diphenyl-β- picrylhydrazyl (DPPH), potassium persulfate, Folin-Ciocalteu’s reagent, and gallic acid were purchased from Sigma-Aldrich (St. Louis, MO). The SIRT1 Direct Fluorescent Screening Assay Kit was from ABNOVA (Cat # KA1366, Taiwan).

### Algae material

The currently accepted scientific names of algae species used in this study are given according to Algaebase^66^ and following the rules of the International Code of Nomenclature for algae, fungi, and plants (ICN)^67^.

To make easier the discussion of the results, the species were divided based on their size and morphology into two typical algal groups, microalgae and macroalgae, which were classified as follows: green algae (Chlorophyta), red algae (Rhodophyta), and brown algae (Ochrophyta). The cyanobacteria were also included in the green algae group as commonly considered for commercial purposes (Table 4). Dried samples of twenty-four commercially available algae species (Table 4) were purchased in local stores. Three packs, from different production batches, were acquired for each specie. The product of each pack was homogenized with a ball mill (Retsch GmbH & Co., KG, Germany), and the powdered samples were stored in sealed plastic vacuum bags at ambient temperature under dry and dark conditions and analyzed within one month. Additionally, fresh samples of twelve macroalgal species were collected during the 23rd–25th August 2017 (summer season) on the Atlantic coast of northern Spain (Comillas – Cantabria, 43° 23 N and 4° 17 W) (Table 5). Sample collections were done at the same rocky shore site in the intertidal and subtidal (1 m depth) zones during low water. The samples of adult fresh macroalgae were selected and carefully washed with fresh seawater to remove sand or remaining debris, and any epiphytes or animals attached to the algal surface. They were then wrapped in sterile cloths moistened with seawater and kept dark and cool with ice packs (< 15 °C) to preserve the algae alive and healthy until transport to the laboratory. Then they were dried in a cabinet laboratory dryer with air circulation at a constant temperature of 40 °C for 72 h approximately (until a constant weight was achieved). The dried samples were milled as previously described, and the powdered samples were stored in sealed plastic vacuum bags at ambient temperature under dry and dark conditions and analyzed within one month.

**Table 4:** Product information retrieved from the label in commercial microalgae and macroalgae (or seaweeds).

Abbreviation	Name	Currently accepted scientific names	Classification	Sample type	Origin
Po	Nori	*Porphyra* sensu lato	Rhodophyta (Macroalgae)	Dried	Spain
Gp	Fresh Gigartina	*Gigartina pistillata*	Rhodophyta (Macroalgae)	Fresh	Spain
Chc	Irish moss	*Chondrus crispus*	Rhodophyta (Macroalgae)	Dried	Spain
Ei	Green Aonori (*Enteromorpha intestinalis*)	***Ulva intestinalis***	Chlorophyta (Macroalgae)	Fresh	Japan
Ms	Irish Moss	*Mastocarpus stellatus*	Rhodophyta (Macroalgae)	Dried	Spain
Pp	Dulse	***Palmaria palmata***	Rhodophyta (Macroalgae)	Dried	Spain
Afa	Klamath eco	***Aphanizomenon flos-aquae***	Cyanobacteria (Microalgae)	Dried	Spain
Cl	Green Caviar	*Caulerpa lentillifera*	Chlorophyta (Macroalgae)	Dried	Unknown
Csp	Barnacle seaweed	*Codium* sp	Chlorophyta (Macroalgae)	Dried	Spain
Ds	Dunaliella	***Dunaliella salina***	Chlorophyta (Microalgae)	Dried	Spain
Sp	Spìrulina (*Spirulina platensis*)	***Arthrospira platensis***	Cyanobacteria (Microalgae)	Dried	Spain
Cv	Chlorella	***Chlorella vulgaris***	Chlorophyta (Microalgae)	Dried	Spain
Tch	Holofit TetraSod capsules	***Tetraselmis chui***	Chlorophyta (Microalgae)	Dried	Spain
Aup	Holofit Chlorella (*Chlorella pyrenoidosa*)	***Auxenochlorella pyrenoidosa***	Chlorophyta (Microalgae)	Dried	Spain
Nsp	Nannochloropsis	***Nannochloropsis *sp.**	Ochrophyta (Microalgae)	Dried	Spain
Ul	Sea Lettuce	***Ulva lactuca***	Chlorophyta (Macroalgae)	Dried	Spain
An	*Ascophyllum nodosum*	***Ascophyllum nodosum***	Ochrophyta, *Phaeophyceae* (Macroalgae)	Dried	Spain
Sf	Iziki seaweed (*Hizikia fusiformis*)	***Sargassum fusiforme***	Ochrophyta, *Phaeophyceae* (Macroalgae)	Dried	Spain
Eb	Arame	***Eisenia bicyclis***	Ochrophyta, *Phaeophyceae* (Macroalgae)	Dried	Unknown
Lo	Kombu bio	***Laminaria ochroleuca***	Ochrophyta, *Phaeophyceae* (Macroalgae)	Dried	Spain
He	Sea Spaguetti bio	***Himanthalia elongata***	Ochrophyta, *Phaeophyceae* (Macroalgae)	Dried	Spain
Up	Wakame	***Undaria pinnatifida***	Ochrophyta, *Phaeophyceae* (Macroalgae)	Dried	Spain
Oa	Odontella capsules	***Odontella aurita***	Bacillariophyta (Microalga)	Dried	France
Fv	Fucus capsules	***Fucus vesiculosus***	Ochrophyta, *Phaeophyceae* (Macroalgae)	Dried	Spain

The algae species included in the novel food catalogue, the union list of authorized novel foods, and official member states’ lists of food and food supplements in Europe are highlighted in bold.

**Table 5:** Sampling location of macroalgae collected from wild populations on Spain's north-western coast.

Abbreviation	Currently accepted scientific names	Class	Location
Gc	*Gelidium corneum*	*Rhodophyceae*	Trasvia, Comillas (Cantabria)
*Pc*	*Plocammium cartilagineum*	*Rhodophyceae*	Trasvia, Comillas (Cantabria)
*Cc*	*Centroceras clavulatum*	*Rhodophyceae*	Comillas' beach (Cantabria)
*Hi*	*Halopithys incurva*	*Rhodophyceae*	Trasvia, Comillas (Cantabria)
*Cd*	*Codium decorticatum*	*Chlorophyceae*	Trasvia, Comillas (Cantabria)
*Bb*	*Bifurcaria bifurcata*	*Phaeophyceae*	Trasvia, Comillas (Cantabria)
*Fg*	*Fucus guiryi*	*Phaeophyceae*	Comillas' beach (Cantabria)
*Pc*	*Pelvetia canaliculata*	*Phaeophyceae*	Comillas' beach (Cantabria)
*Hs*	*Halopteris scoparia*	*Phaeophyceae*	Trasvia, Comillas (Cantabria)
*Gb*	*Gongolaria baccata*	*Phaeophyceae*	Trasvia, Comillas (Cantabria)
*Cs*	*Cladostephus spongiosus*	*Phaeophyceae*	Comillas' beach (Cantabria)
*Es*	*Ericaria selaginoides*	*Phaeophyceae*	Trasvia, Comillas (Cantabria)

### Preparation of the enzymatic protein hydrolysates (EPH)

With minor modifications, the procedure used to obtain the EPH fractions was based on a previously described method^68^. In brief, 0.5 g of dried milled algae powder was suspended in 10 mL of milli-Q water, stirred gently with a Reax 2 shaker (Heidolph Instruments GmbH & CO. KG, Schwabach-Germany) for 3 h at 4 °C and then centrifuged at 4000*g* for 15 min at 4 °C (mod. 5430R, Eppendorf AG, Hamburg—Germany). Water-soluble proteins were precipitated by adjusting the pH of the supernatant fraction to 3.5 with HCl 0.1 N and keeping the sample at 4 °C for 30 min. After centrifuging the sample at 4000*g* for 15 min at 4 °C, the pellets (protein isolate) were collected and freeze-dried with a LyoMicron (Cool Vacuum Technologies SL, Barcelona, Spain). Three independent extracts were obtained for each algae specie.

EPH fractions were obtained by enzymatic hydrolysis of the dried pellets by using the enzyme Alcalase® (Merck KGaA, Darmstadt, Germany) following Pérez-Míguez et al.^68^. Pellets (50 mg) were dissolved, with the help of an ultrasonic bath (J.P. Selecta, BCN, Spain), in 10 mL of 5 mM borate buffer, pH = 8.5 (final concentration of the EPH fractions = 5 mg/mL). Then, Alcalase® was added at an enzyme/substrate ratio of 0.15 AU per gram of dried sample. The digestion was performed at 50 °C for 4 h with agitation (700 rpm) in a Thermomixer Compact (Eppendorf AG, Hamburg, Germany). The digestion/reaction was stopped by heating (100 °C for 10 min) and the solution was centrifuged for 10 min at 6000*g* (mod. 5430R; Eppendorf AG, Hamburg Germany). Finally, the supernatants (EPH) were collected and stored at -80 ºC until analysis.

### Determination of protein content and protein recovery

The protein content of the dried algae samples and enzymatic protein hydrolysates (EPH) was determined following a previously published method^69^, with minor modifications. In brief, 5 mg of dried sample or five microliters of EPH were re-suspended by vortexing in 200 L of 24% (w/v) TCA, incubated in a water bath for 15 min at 95 C, and then allowed to cool at room temperature. After the addition of 600 L of MilliQ water and mixing, the samples were centrifuged at 15,000*g* for 20 min at 4 C (Microcentrifuge 5415 R, Eppendorf AG, Hamburg, Germany) and their supernatants discarded. The pellets were resuspended in 0.5 mL of Lowry Reagent D and incubated for 10 min at 55 C. Samples were then cooled at room temperature, centrifuged at 15,000*g* for 20 min, and the supernatant retained. For protein quantification, a stock of Lowry Reagent D was prepared daily in a 48:1:1 ratio (v/v/v) of Lowry Reagents A, B, and C. A suitable volume (up to 50 μL) of the protein extract was added into a 1.5 mL microfuge tube, together with 950 L of Lowry Reagent D, followed by immediate mixing. After incubation for 10 min at room temperature, 0.1 mL of 0.2 N Folin-Ciocalteu phenol reagent was added to each tube and mixed immediately. After 30 min at room temperature, absorbance was read at 600 nm (1800 UV–Vis spectrophotometer, Shimadzu Co., Madison, WI) using UVProbe™ software (Shimadzu Co., Madison, WI). Calibration curves were prepared for each assay with a bovine serum albumin (BSA) stock solution (200 mg/mL) and using a polynomial line of best fit generated in Microsoft Excel 365. Analysis was performed in triplicate and expressed in g / 100 g d.w.

The recovery of the protein fraction (RP) was determined using the Eq. (1):

1 $$RP = \frac{{P_{1} }}{{P_{2} }} \times 100$$

where P1 is the protein content in algae protein hydrolysates and P2 is the algae protein in dried algal samples.

### Amino acid and peptide quantification in algae hydrolysates

Total amino acids were quantified as previously described^70^. Briefly, 10 mg of EPH fractions were weighted and added with 1 mL of 8 M per chloric acid and maintained 24 h at 110 °C (Precisiterm, J.P. Selecta, BCN, Spain). After cooling at room temperature, the samples were filtered through 0.2 µm membrane syringe filters (GMP filter membranes, Merck KGaA, Darmstadt, Germany), derivatized with a methanol: water: TEA: PITC solution (7:1:1:1, v:v:v:v) and evaporated under nitrogen. Then, 24 µL of mobile phase B and 226 µL of mobile phase A to the derivatized samples centrifuged at 11,000×*g* for 5 min and filtered through a Single Step Standard Filter Vials (Thomson Instrument Company, CA, USA). Four µL of the sample were injected into the chromatographic system, consisting of an Acquity UPLC® equipped with a PDA detector, an electrospray (ESI) as a source of ionization operated in the positive mode, and a TQD triple quadrupole mass spectrometer (Waters, Milford, MA, USA). The data were acquired with MassLynx v.4.1 software (Waters, Milford, MA, USA). Chromatographic separation was carried out on a BEH-C_18_, 1.7 µm, 100 mm × 2.1 mm i.d. column (Waters, Milford, MA, USA). Peak identity was confirmed by comparing their retention times, UV spectra, MS and MS/MS spectra with the corresponding data obtained from pure standards.

The total hydrophobic amino acid content was determined as the sum of phenylalanine, leucine, isoleucine, tyrosine, valine, methionine, and proline concentrations as previously described^71^.

Peptide content was estimated by using the O-phthaldialdehyde method (OPA)^72^ with minor modifications. Every day, a fresh OPA solution was prepared by combining: (1) solution A (7.62 g sodium tetrahydroborate and 200 mg sodium dodecyl sulfate dissolved in 150 mL of deionized water), (2) solution B (160 mg of OPA dissolved in 4 mL of ethanol (96%), and (3) solution C (400 L of -mercaptoethanol, adjusted to a final volume of μL of the EPH fraction was mixed with 270 μL of OPA reagent in a 96-well plate. The mixture was incubated at room temperature for 2 min, and then the absorbance was measured at 340 nm using a microplate reader system (Varioskan, Thermo Scientific, MA, USA). The analysis was carried out in triplicate for each of the 3 extracts from each algae specie (n = 9). The peptide content, expressed as mg of glutathione (GSH) per mL of EPH, was calculated by creating a calibration curve with glutathione solutions in the range of 0.1–5.0 mg/mL.

### Trolox equivalent antioxidant capacity

The trolox equivalent antioxidant capacity (TEAC) assay was carried out according to Li et al.^73^. In the TEAC assay, antioxidant action by hydrogen atom transfer (HAT) as well as single electron transfer (SET) is measured. For the assay, an ABTS· + radical cation was generated by preparing a solution of 7 mM ABTS and 2.45 mM potassium persulfate in milliQ-water. The reaction mixture was kept in the dark for 16 h at room temperature and used within two days. The ABTS· + solution was diluted with deionized water to give an absorbance of 0.700 ± 0.050 at 734 nm. 50 μL of EPH fraction was mixed with 1.9 mL of diluted ABTS· + solution. After incubating for 10 min at room temperature, the absorbance was measured with a UV-1800 spectrophotometer (Shimadzu Corp., Kyoto, Japan) at 734 nm. The antioxidant capacity of the tested samples was calculated by determining the decrease in absorbance at different concentrations by using the Eq. (2):

2 $$\%\,TEAC = \left[ {\frac{{Abs_{control} - Abs_{sample} }}{{Abs_{control} }}} \right] \times 100$$

where Abscontrol and Abssample are the absorbances of the ABTS· + and the tested samples, respectively.

### DPPH free radical scavenging activity

The DPPH (1,1-diphenyl-2-picrylhydrazyl) radical scavenging activity was determined according to the method of Zhang et al.^74^, with slight modifications. Briefly, 100 μL of EPH fraction were mixed with 100 μL of 0.16 mM DPPH methanolic solution. This mixture was vortexed for 1 min, kept for 30 min in the dark, and then the absorbance was read at 517 nm in an automated microplate reader (Sunrise-Elisa Reader, Tecan, Swiss). The radical scavenging activity was calculated using the Eq. (3):

3 $$\%\,DPPH = \left[ {\frac{{Abs_{control } - \left( {Abs_{sample} - Abs_{blank} } \right)}}{{Abs_{control} }}} \right] \times 100$$

where the Abs_control_ is the absorbance of the control (DPPH without sample), the Abs_sample_ is the absorbance of the sample (sample plus DPPH solution), and the Abs_blank_ is the absorbance of the sample blank (Sample without the DPPH solution).

### SIRT1 direct fluorescence assay

SIRT1 activity modulation was assessed by using a Direct Fluorescent Screening Assay Kit (SIRT1 Direct Fluorescent Screening Assay Kit, Cat # KA1366, ABNOVA, Taiwan) following the instructions of the provider (Figures S1 and S2, *Supplementary material*). The formation of the final fluorescent product was detected using a Varioskan (Varioskan, Thermo Scientific, MA, USA) with an excitation wavelength of 360 nm and an emission wavelength of 465 nm.

For each EPH fraction, the % inhibition/activation was calculated following the Eq. (4), and the fold activation was calculated following the instructions of the provider by using the Eq. (5):

4 $$\%\,Inhibition/Activation = \left[ {\frac{{Initial\,Activity\,fluorescence_{control} - Sample\,fluorescence}}{{Initial\,Activity\,fluorescence_{control} }}} \right] \times 100$$

5 $$Fold\,Activation = \frac{Sample\,fluorescence}{{Initial\,Activity\,fluorescence_{control} }}$$

where “Initial Activity fluorescence _control_” is the fluorescence obtained in wells with SIRT1 dissolved in assay buffer and solvent;

“Sample fluorescence” is the fluorescence from wells with SIRT1 dissolved in buffer assay and solvent plus samples.

To perform the Pearson´s correlation test and the principal component analysis, negative values of SIRT1 activity were scaled and normalized into a range of 0 and 1, according to Teknomo^75^.

### Statistical analysis

All results were expressed as the mean ± standard deviation.

A Kruskal–Wallis non-parametric test and Mann–Whitney pairwise comparisons of median values were carried out to evidence significant differences between groups (*p* < 0.05). Principal component analysis (PCA) and Pearson's correlation coefficients (PCC) were used. Statistical analyses were performed with the software Minitab® version 19.2, 2019 (Minitab Inc., State College, PA, USA), and RStudio version 2022.02.3, Build 492 (RStudio: Integrated Development for R. RStudio, Inc., Boston, MA, USA).

## Supplementary Information

Supplementary Information.

## Data Availability

All data that support the findings of this study are provide in the manuscript and supplementary file.
