# Supplementary material for: Taurine, homotaurine, GABA and hydrophobic amino acids content influences “in vitro” antioxidant and SIRT1 modulation activities of enzymatic protein hydrolysates from algae
Source: Sci Rep. 2022 Dec 2;12:20832. doi: 10.1038/s41598-022-25130-4 (PMC9718854; doi:10.1038/s41598-022-25130-4)
Supplement: Supplementary file 1 — Supplementary Information. [file 41598_2022_25130_MOESM1_ESM.docx]

**Taurine, Homotaurine, GABA and hydrophobic amino acids content influences “in vitro” antioxidant and SIRT1 modulation activities** **of enzymatic protein hydrolysates from algae**

Carlos Terriente-Palacios ^a, b^, Susana Rubino Campoy ^a^, Maria Hortós ^a^, César Peteiro ^c^. Massimo Castellari ^*, a^

^a^ IRTA Food Industries – Finca Camps I Armet s/n, 17121 – Monells, Girona (Spain)

Tel. 972 63 00 52 Ext.1414

^b^ Escuela Internacional de Doctorado UNED - Calle Bravo Murillo 38, 28015 Madrid, Spain

^c^ Spanish Institute of Oceanography of the Spanish National Research Council (IEO, CSIC), Oceanographic Center of Santander, Marine Culture Units “El Bocal”, Seaweeds Center, Barrio Corbanera s/n., 39012 Monte, Santander (Spain)

*Corresponding author: e-mail Massimo.Castellari@irta.cat

**
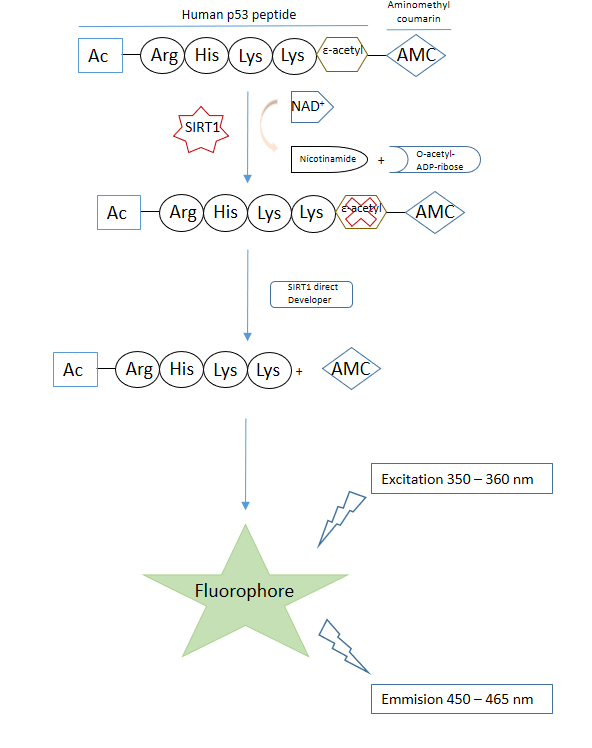
**

**Figure S1**. Principle of the SIRT1 Fluorescence screening assay used in this study.

**
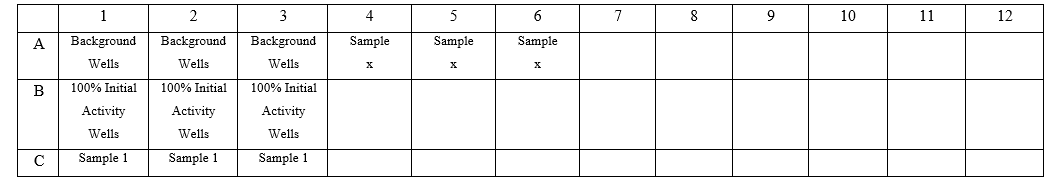

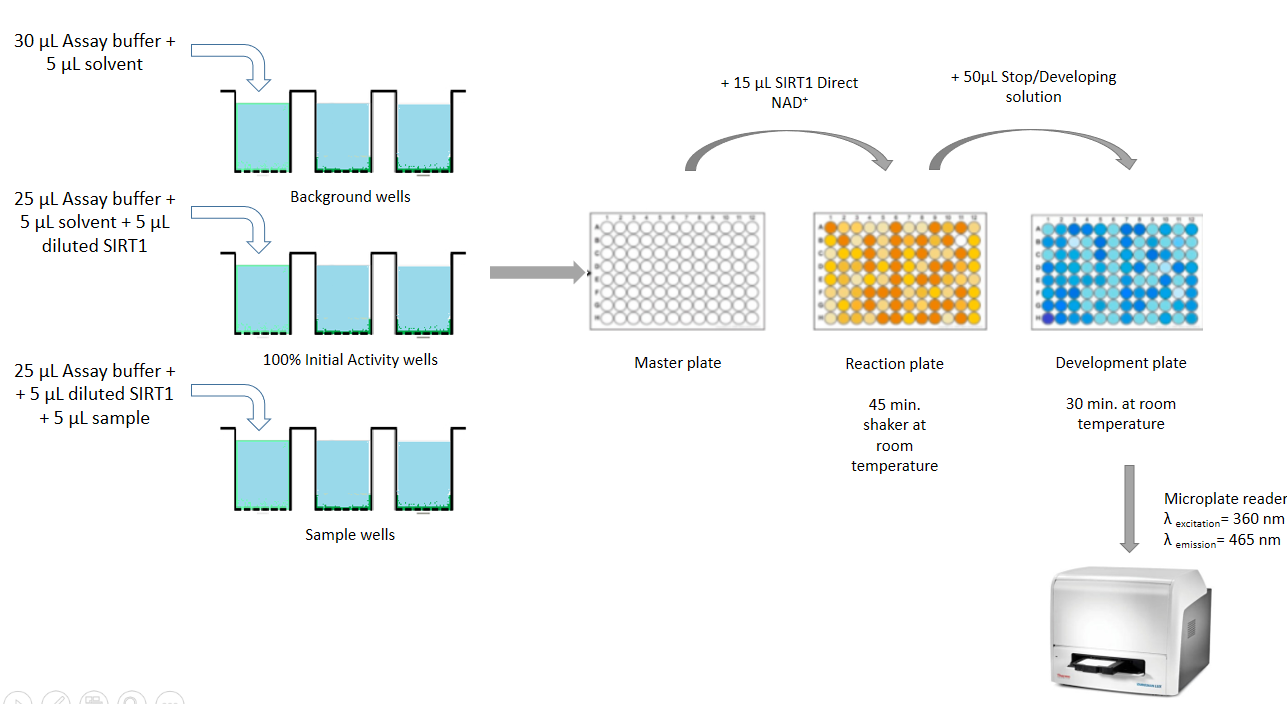
**

**Figure S2.** Scheme of the Assay protocol of ABNOVA SIRT1 Fluorescence kit.

**Table S1.** Amino acid profiles of 36 algal protein hydrolysates. Data are expressed as g / 100 g protein. Each value is the mean of n = 3 independent determinations ± standard deviation.

| Amino acids | *Porphyra sp.* | *Gigartina pistillata* | *Chondrus crispus* | *Enteromorpha intestinalis* | *Mastocarpus stellatus* | *Palmaria palmata* | *Gelidium corneum* | *Plocamium cartilagineum* | *Centroceras clavulatum* | *Halopithys incurva* |
| --- | --- | --- | --- | --- | --- | --- | --- | --- | --- | --- |
|  |  |  |  |  |  |  |  |  |  |  |
| His | 1.05 ± 0.15 | 0.41 ± 0.03 | 0.87 ± 0.18 | 1.64 ± 0.65 | 1.38 ± 0.34 | 1.46 ± 0.21 | 0.48 ± 0.05 | 1.85 ± 0.08 | 1.58 ± 0.06 | 1.33 ± 0.06 |
| Thr | 4.35 ± 0.21 | 4.70 ± 0.04 | 1.88 ± 0.02 | 2.72 ± 0.13 | 2.23 ± 0.01 | 4.23 ± 0.65 | 5.40 ± 0.06 | 5.23 ± 1.41 | 4.53 ± 0.71 | 3.29 ± 0.19 |
| Lys | 4.45 ± 0.32 | 6.23 ± 0.25 | 11.1 ± 0.87 | 5.66 ± 0.75 | 8.29 ± 0.08 | 10.7 ± 1.36 | 5.07 ± 1.96 | 5.08 ± 0.32 | 5.31 ± 1.94 | 6.45 ± 1.54 |
| Met | 1.02 ± 0.07 | 0.44 ± 0.10 | 0.09 ± 0.01 | 0.56 ± 0.08 | 0.55 ± 0.07 | 0.86 ± 0.09 | 3.51 ± 0.04 | 2.10 ± 0.12 | 2.67 ± 0.01 | 2.89 ± 0.84 |
| Val | 4.03 ± 0.21 | 6.94 ± 2.12 | 3.22 ± 0.72 | 9.97 ± 0.34 | 3.28 ± 0.69 | 4.50 ± 0.86 | 13.9 ± 1.30 | 4.60 ± 0.25 | 8.14 ± 0.91 | 7.75 ± 1.73 |
| Ileu | 2.98 ± 0.34 | 1.87 ± 0.17 | 4.09 ± 0.46 | 6.09 ± 1.39 | 2.84 ± 0.36 | 4.37 ± 0.61 | 7.20 ± 0.89 | 4.60 ± 0.23 | 6.62 ± 0.89 | 7.09 ± 0.60 |
| Leu | 5.23 ± 0.14 | 3.63 ± 0.58 | 5.58 ± 0.90 | 10.7 ± 1.18 | 4.45 ± 0.41 | 5.20 ± 0.48 | 10.6 ± 0.44 | 8.84 ± 1.70 | 14.4 ± 2.52 | 16.6 ± 1.79 |
| Phe | 2.02 ± 0.11 | 2.32 ± 0.89 | 3.22 ± 0.26 | 5.52 ± 0.15 | 3.55 ± 0.52 | 4.75 ± 1.73 | 5.20 ± 0.62 | 5.81 ± 0.95 | 6.07 ± 1.78 | 4.75 ± 0.31 |
| ∑EAA | 25.1 | 26.5 | 30.0 | 43.5 | 26.6 | 29.4 | 49.5 | 42.0 | 46.6 | 47.3 |
| Hyptau | 0.03 ± 0.01 | nd | nd | nd | 0.01 ± 2.12 | 0.01 ± 2.36 | nd | nd | nd | nd |
| Hyp | 0.02 ± 0.00 | nd | 0.03 ± 0.00 | 0.05 ± 0.00 | 0.10 ± 0.03 | 0.10 ± 0.02 | 0.03 ± 0.01 | 0.03 ± 0.01 | 0.03 ± 0.01 | 0.04 ± 0.01 |
| Tau | 0.35 ± 0.02 | 0.38 ± 0.02 | 0.24 ± 0.01 | 0.26 ± 0.01 | 0.14 ± 0.01 | 0.06 ± 0.01 | 0.03 ± 0.01 | 0.04 ± 0.01 | 0.02 ± 0.04 | 0.04 ± 0.01 |
| Htau | 0.06 ± 0.01 | 0.05 ± 0.00 | 0.04 ± 0.00 | 0.04 ± 0.01 | 0.05 ± 0.01 | 0.02 ± 0.01 | 0.01 ± 0.01 | 0.02 ± 0.01 | 0.004 ± 0.01 | 0.02 ± 0.01 |
| Arg | 4.97 ± 0.12 | 3.39 ± 0.50 | 2.54 ± 0.07 | 2.27 ± 0.07 | 2.29 ± 0.14 | 4.94 ± 0.63 | 6.58 ± 1.20 | 5.27 ± 0.38 | 7.00 ± 1.02 | 6.78 ± 0.72 |
| Ser | 13.3 ± 0.24 | 2.21 ± 0.11 | 6.88 ± 1.17 | 3.50 ± 0.69 | 2.35 ± 1.01 | 10.7 ± 1.50 | 4.01 ± 0.36 | 6.00 ± 1.39 | 4.16 ± 0.21 | 4.68 ± 0.16 |
| Gly | 6.81 ± 0.34 | 2.17 ± 0.26 | 7.97 ± 0.19 | 4.17 ± 0.62 | 5.34 ± 0.15 | 10.6 ± 1.27 | 4.55 ± 0.72 | 6.75 ± 0.14 | 4.16 ± 1.51 | 4.68 ± 1.25 |
| Asp | 5.32 ± 0.18 | 2.94 ± 0.52 | 4.23 ± 1.50 | 6.73 ± 0.41 | 6.47 ± 1.69 | 9.81 ± 0.94 | 8.62 ± 1.08 | 13.4 ± 0.55 | 9.80 ± 1.15 | 8.93 ± 1.03 |
| Glu | 10.0 ± 0.33 | 5.09 ± 1.54 | 4.85 ± 1.62 | 12.1 ± 2.02 | 5.86 ± 0.51 | 8.01 ± 1.60 | 11.4 ± 0.26 | 12.8 ± 0.46 | 10.0 ± 1.80 | 8.55 ± 0.61 |
| Cys | 1.15 ± 0.04 | nd | 3.22 ± 1.76 | 0.29 ± 0.64 | 1.32 ± 0.51 | 2.98 ± 0.59 | 0.03 ± 0.00 | 1.17 ± 0.38 | 1.30 ± 0.09 | 0.97 ± 0.01 |
| Pro | 2.72 ± 0.24 | 5.41 ± 1.39 | 4.78 ± 0.95 | 7.81 ± 0.15 | 3.14 ± 0.05 | 7.67 ± 1.66 | 4.89 ± 0.94 | 4.01 ± 0.33 | 3.49 ± 0.87 | 5.11 ± 0.33 |
| Ala | 2.31 ± 0.32 | 7.30 ± 0.16 | 1.48 ± 0.91 | 7.52 ± 0.73 | 5.93 ± 1.15 | 6.38 ± 0.42 | 5.41 ± 0.04 | 7.99 ± 0.61 | 5.58 ± 0.32 | 6.27 ± 0.83 |
| GABA | 1.25 ± 0.08 | 1.14 ± 0.01 | 1.05 ± 0.11 | 0.88 ± 0.01 | 0.82 ± 2.74 | 0.35 ± 0.02 | 0.39 ± 0.01 | 0.29 ± 0.06 | 0.19 ± 0.05 | 0.37 ± 0.03 |
| Tyr | 3.55 ± 0.17 | 0.04 ± 0.01 | 0.82 ± 0.02 | 4.15 ± 0.06 | 2.19 ± 0.17 | 1.01± 0.07 | 2.75 ± 0.22 | 3.05 ± 0.04 | 3.49 ± 0.09 | 2.73 ± 0.90 |
| ∑NEAA | 51.9 | 30.1 | 38.1 | 56.2 | 36.0 | 62.7 | 48.7 | 60.9 | 49.2 | 49.2 |
| Total Amino acids | 77.0 | 56.7 | 68.2 | 99.1 | 62.6 | 98.9 | 100 | 99.0 | 95.5 | 99.4 |

| Amino acids | *Aphanizomenon flos-aquae* | *Caulerpa lentillifera* | *Codium sp.* | *Dunaliella salina* | *Athrospira platensis* | | | *Chlorella vulgaris* | | | *Tetraselmis chui* | | | *Auxenochlorella pyrenoidosa* | | | *Nanochloropsis sp.* | | | *Ulva lactuca* | | |
| --- | --- | --- | --- | --- | --- | --- | --- | --- | --- | --- | --- | --- | --- | --- | --- | --- | --- | --- | --- | --- | --- | --- |
|  |  |  |  |  |  |  |  |  |  |  |  |  |  |  |  |  |  |  |  |  |  |  |
| His | 1.18 ± 0.77 | 1.40 ± 0.27 | 1.00 ± 0.06 | 0.47 ± 0.03 | 1.55 ± 0.19 | | | 3.00 ± 0.22 | | | 2.07 ± 0.19 | | | 2.56 ± 0.18 | | | 0.59 ± 0.06 | | | 1.13 ± 0.15 | | |
| Thr | 5.08 ± 0.66 | 1.62 ± 0.06 | 0.77 ± 0.17 | 1.59 ± 0.13 | 1.57 ± 0.13 | | | 4.55 ± 0.83 | | | 2.43 ± 1.22 | | | 5.08 ± 1.15 | | | 3.02 ± 0.79 | | | 2.94 ± 0.77 | | |
| Lys | 5.85 ± 0.48 | 0.31 ± 0.02 | 1.78 ± 0.03 | 2.62 ± 0.77 | 5.57 ± 0.98 | | | 10.3 ± 1.02 | | | 3.48 ± 0.83 | | | 7.55 ± 1.06 | | | 2.30 ± 0.84 | | | 3.47 ± 1.08 | | |
| Met | 1.00 ± 0.11 | 1.15 ± 0.16 | 0.68 ± 0.10 | 0.03 ± 0.00 | 2.48 ± 0.15 | | | 1.26 ± 0.25 | | | 0.60 ± 0.15 | | | 0.56 ± 0.14 | | | 0.13 ± 0.01 | | | 1.84 ± 0.08 | | |
| Val | 1.52 ± 0.14 | 6.33 ± 0.87 | 2.35 ± 0.45 | 1.73 ± 0.14 | 5.24 ± 0.96 | | | 2.80 ± 0.18 | | | 4.31 ± 1.23 | | | 4.30 ± 1.11 | | | 4.62 ± 1.09 | | | 2.40 ± 1.15 | | |
| Ileu | 5.90 ± 0.01 | 3.42 ± 0.53 | 1.16 ± 0.06 | 7.65 ± 0.82 | 4.27 ± 0.80 | | | 4.44 ± 0.93 | | | 1.86 ± 1.20 | | | 3.13 ± 0.09 | | | 11.9 ± 1.21 | | | 2.16 ± 0.99 | | |
| Leu | 7.28 ± 1.65 | 5.79 ± 0.72 | 1.92 ± 0.55 | 6.34 ± 0.84 | 7.36 ± 1.00 | | | 6.94 ± 0.82 | | | 3.51 ± 0.73 | | | 5.08 ± 1.02 | | | 3.86 ± 0.75 | | | 4.02 ± 1.17 | | |
| Phe | 5.60 ± 0.79 | 3.30 ± 0.87 | 1.35 ± 0.33 | 1.45 ± 0.80 | 2.21 ± 0.18 | | | 5.38 ± 0.18 | | | 0.01 ± 0.00 | | | 4.22 ± 0.81 | | | 0.32 ± 0.04 | | | 2.46 ± 0.04 | | |
| ∑EAA | 33.4 | 23.3 | 11.0 | 21.9 | 30.3 | | | 38.8 | | | 18.3 | | | 32.5 | | | 26.8 | | | 20.4 | | |
| Hyptau | nd | nd | nd | nd | nd | | | nd | | | nd | | | 0.01 ± 0.01 | | | nd | | | nd | | |
| Hyp | 0.02 ± 0.00 | 0.08 ± 0.01 | 0.02 ± 0.01 | 0.02 ± 0.00 | 1.07 ± 0.13 | | | 0.36 ± 0.01 | | | 0.04 ± 0.00 | | | 0.37 ± 0.05 | | | 0.02 ± 0.01 | | | 0.82 ± 0.01 | | |
| Tau | 0.24 ± 0.01 | 0.39 ± 0.02 | 0.38 ± 0.01 | 0.35 ± 0.00 | 0.35 ± 0.03 | | | 0.31 ± 0.00 | | | 0.25 ± 0.05 | | | 0.27 ± 0.00 | | | 0.15 ± 0.06 | | | 0.03 ± 0.00 | | |
| Htau | 0.04 ± 0.00 | 0.06 ± 0.00 | 0.05 ± 0.00 | 0.04 ± 0.00 | 0.04 ± 0.00 | | | 0.04 ± 0.00 | | | 0.04 ± 0.00 | | | 0.04 ± 0.00 | | | 0.03 ± 0.00 | | | 0.02 ± 0.00 | | |
| Arg | 5.35 ± 0.42 | 2.15 ± 0.21 | 1.03 ± 0.12 | 0.37 ± 0.03 | 11.6 ± 0.76 | | | 10.3 ± 1.04 | | | 1.75 ± 0.06 | | | 6.16 ± 0.98 | | | 1.50 ± 0.92 | | | 2.65 ± 0.99 | | |
| Ser | 4.72 ± 0.35 | 3.52 ± 0.92 | 1.04 ± 0.03 | 1.62 ± 0.19 | 5.81 ± 1.06 | | | 5.47 ± 1.05 | | | 3.20 ± 0.99 | | | 3.41 ± 0.07 | | | 2.75 ± 0.18 | | | 4.55 ± 1.20 | | |
| Gly | 5.35 ± 0.34 | 3.87 ± 0.42 | 1.78 ± 0.16 | 3.28 ± 0.04 | 7.45 ± 1.06 | | | 8.97 ± 1.17 | | | 2.67 ± 1.18 | | | 8.97 ± 0.82 | | | 7.62 ± 0.79 | | | 1.75 ± 0.03 | | |
| Asp | 7.47 ± 1.76 | 13.0 ± 1.65 | 1.35 ± 0.18 | 4.21 ± 0.73 | 13.1 ± 0.99 | | | 12.1 ± 0.77 | | | 5.48 ± 1.25 | | | 10.7 ± 1.01 | | | 8.43 ± 1.13 | | | 2.97 ± 0.94 | | |
| Glu | 11.3 ± 0.45 | 11.6 ± 0.67 | 4.35± 0.39 | 5.78 ± 1.04 | 19.9 ± 0.99 | | | 14.5 ± 1.09 | | | 6.05 ± 1.09 | | | 12.5 ± 0.85 | | | 11.5 ± 0.91 | | | 2.35 ± 0.88 | | |
| Cys | 0.02 ± 0.00 | 0.10 ± 0.06 | 0.17 ± 0.07 | 1.10 ±0.01 | 1.75 ± 0.11 | | | 1.75 ± 0.24 | | | 0.31 ± 0.02 | | | 0.29 ± 0.04 | | | 0.01 ± 0.00 | | | 1.26 ± 0.12 | | |
| Pro | 5.00 ± 1.64 | 5.71 ± 1.16 | 1.39± 0 .35 | 3.78 ± 0.74 | 3.18 ± 0.93 | | | 3.61 ± 1 .14 | | | 1.58 ± 0.31 | | | 5.36 ± 1.09 | | | 4.91 ± 0.79 | | | 1.87 ± 0.07 | | |
| Ala | 6.95 ± 0.34 | 8.03 ± 0.01 | 1.68 ± 0.47 | 7.03 ± 0.55 | 10.7 ± 0.82 | | | 9.85 ± 1.04 | | | 5.37 ± 0.87 | | | 10.0 ± 0.73 | | | 9.59 ± 0.75 | | | 2.18 ± 0.15 | | |
| GABA | 1.05 ± 0.08 | 1.28 ± 0.09 | 1.27 ± 0.05 | 0.91± 0 .03 | 0.83 ± 0.05 | | | 0.94 ± 0.05 | | | 0.63 ± 0.20 | | | 0.72 ± 0.12 | | | 0.49 ± 0.05 | | | 0.37 ± 0.03 | | |
| Tyr | 0.42 ± 0.03 | 2.97 ± 0.20 | 1.00 ± 0.12 | 0.48 ± 0.16 | 1.88 ± 0.12 | | | 2.09 ± 0.06 | | | 1.23 ± 0.11 | | | 2.86 ± 0.05 | | | 1.40 ± 0.19 | | | 1.40 ± 0.22 | | |
| ∑NEAA | 47.9 | 52.8 | 15.5 | 28.9 | 77.7 | | | 70.4 | | | 28.6 | | | 61.7 | | | 48.4 | | | 22.2 | | |
| Total Amino acids | 81.4 | 76.1 | 26.5 | 50.9 | 108 |  |  | 109 |  |  | 46.9 |  |  | 94.3 |  |  | 75.2 |  |  | 42.6 |  |  |

| Amino acids | *Codium decorticatum* | *Ascophyllum nodosum* | *Sargassum fusiforme* | *Eisenia byciclis* | *Laminaria ochroleuca* | *Himanthalia elongata* | *Undaria pinnatifida* | *Odonella aurita* | *Fucus vesiculosus* | *Bifurcaria bifurcata* |
| --- | --- | --- | --- | --- | --- | --- | --- | --- | --- | --- |
|  |  |  |  |  |  |  |  |  |  |  |
| His | 0.55 ± 0.15 | 0.53 ± 0.13 | 0.21 ± 0.08 | 0.50 ± 0.01 | 3.12 ± 0.48 | 5.66 ± 0.69 | 0.90 ± 0.01 | 0.74 ± 0.11 | 1.83 ± 0.50 | 1.91 ± 0.20 |
| Thr | 0.91 ± 0.03 | 1.08 ± 0.11 | 0.70 ± 0.05 | 0.86 ± 0.14 | 1.61 ± 0.22 | 2.29 ± 0.36 | 9.04 ± 0.57 | 2.73 ± 0.21 | 3.84 ± 0.63 | 5.36 ± 0.35 |
| Lys | 1.67 ± 0.21 | 2.06 ± 0.86 | 0.86± 0.01 | 0.58 ± 0.08 | 3.10 ± 0.44 | 5.05 ± 0.54 | 7.48 ± 0.66 | 12.1 ± 0.32 | 9.53 ± 0.40 | 5.60 ± 0.21 |
| Met | 1.21 ± 0.22 | 0.06 ± 0.01 | 0.69 ± 0.09 | 1.22 ± 0.06 | 0.12 ± 0.02 | 0.35 ± 0.06 | 0.16 ± 0.02 | 0.16 ± 0.06 | 0.14 ± 0.01 | 0.30 ± 0.02 |
| Val | 3.92 ± 1.20 | 1.29 ± 0.12 | 0.52 ± 0.02 | 1.01 ± 0.15 | 5.97 ± 0.33 | 3.53 ± 0.21 | 3.07 ± 0.52 | 2.20 ± 0.37 | 3.86 ± 0.59 | 5.38 ± 0.23 |
| Ileu | 1.88 ± 0.19 | 0.85 ± 0.02 | 0.38 ± 0.12 | 0.80 ± 0.13 | 3.65 ± 0.69 | 0.09 ± 0.01 | 5.07 ± 0.60 | 8.74 ± 0.25 | 1.36 ± 0.24 | 4.34 ± 0.63 |
| Leu | 2.47 ± 0.24 | 1.09 ± 0.06 | 1.83 ± 0.21 | 1.84 ± 0.08 | 6.58 ± 0.23 | 2.30 ± 0.52 | 0.88 ± 0.02 | 3.38 ± 0.70 | 3.41 ± 0.22 | 7.31 ± 0.30 |
| Phe | 1.88 ± 0.24 | 0.48 ± 0.18 | 0.77 ± 018 | 0.77 ± 0.06 | 0.78 ± 0.06 | 0.04 ± 0.01 | 3.51 ± 0.68 | 2.86 ± 0.44 | 1.22 ± 0.34 | 5.10 ± 0.27 |
| ∑EAA | 14.5 | 7.44 | 5.96 | 7.58 | 24.9 | 19.3 | 30.1 | 32.9 | 25.2 | 35.3 |
| Hyptau | nd | nd | nd | nd | nd | nd | nd | nd | nd | nd |
| Hyp | 0.03 ± 0.01 | 0.02 ± 0.01 | 0.03 ± 0.01 | 0.02 ± 0.01 | 0.04 ± 0.01 | 0.39 ± 0.01 | 0.04 ± 0.00 | 0.02 ± 0.00 | 0.10 ± 0.01 | 0.14 ± 0.00 |
| Tau | 0.009 ± 0.000 | 0.19 ± 0.00 | 0.25 ± 0.00 | 0.23 ± 0.00 | 0.25 ± 0.01 | 0.22 ± 0.01 | 0.19 ± 0.05 | 0.19 ± 0.01 | 0.21 ± 0.01 | 0.001 ± 0.000 |
| Htau | 0.007 ± 0.00 | 0.045 ± 0.00 | 0.035 ± 0.00 | 0.033 ± 0.00 | 0.045 ± 0.00 | 0.045 ± 0.00 | 0.038 ± 0.00 | 0.038 ± 0.00 | 0.031 ± 0.00 | 0.008 ± 0.00 |
| Arg | 1.35 ± 0.02 | 0.28 ± 0.01 | 1.25 ± 0.08 | 0.98 ± 0.01 | 1.78 ± 0.01 | 0.88 ± 0.11 | 6.93 ± 0.13 | 0.04 ± 0.00 | 3.00 ± 0.22 | 5.10 ± 0.42 |
| Ser | 1.62 ± 0.08 | 0.88 ± 0.07 | 0.61 ± 0.03 | 0.95 ± 0.08 | 7.45 ± 0.68 | 5.66 ± 0.40 | 9.11 ± 0.48 | 1.46 ± 0.20 | 4.04 ± 0.23 | 4.91 ± 0.71 |
| Gly | 1.93 ± 0.23 | 0.60 ± 0.05 | 0.84 ± 0.09 | 1.22 ± 0.16 | 11.1 ± 0.63 | 1.36 ± 012 | 5.37 ± 0.60 | 11.4 ± 0.60 | 9.53 ± 0.53 | 5.38 ± 0.45 |
| Asp | 1.44 ± 0.18 | 1.55 ± 0.15 | 2.35 ± 0.11 | 2.22 ± 0.31 | 3.26 ± 0.21 | 11.2 ± 0.51 | 7.56 ± 0.28 | 2.20 ± 0.19 | 11.7 ± 0.36 | 17.6 ± 0.51 |
| Glu | 4.00 ± 1.1 | 2.55 ± 0.84 | 3.49 ± 1.20 | 4.64 ± 1.20 | 3.38 ± 0.71 | 6.87 ± 0.38 | 8.37 ± 0.28 | 6.75 ± 0.33 | 11.3 ± 0.47 | 23.3 ± 0.48 |
| Cys | 0.21 ± 0.06 | 0.03 ± 0.00 | 0.48 ± 0.03 | 1.50 ± 0.18 | 3.36 ± 0.29 | 2.12 ± 0.05 | 0.16 ± 0.06 | 0.01 ± 0.00 | 0.05 ± 0.01 | 0.02 ± 0.00 |
| Pro | 3.15 ± 0.51 | 0.63 ± 0.05 | 0.60 ± 0.01 | 0.60 ± 0.01 | 9.48 ± 0.64 | 1.21 ± 0.21 | 1.64 ± 0.25 | 8.08 ± 0.55 | 6.74 ± 0.10 | 4.91 ± 0.21 |
| Ala | 1.62 ± 0.08 | 0.75 ± 0.11 | 1.54 ± 0.17 | 1.16 ± 0.22 | 9.48 ± 0.71 | 10.4 ± 0157 | 2.47 ± 0.33 | 0.65 ± 0.09 | 9.53 ± 0.71 | 12.3 ± 0.41 |
| GABA | 0.18 ± 001 | 0.68 ± 0.02 | 0.72 ± 0.01 | 0.74 ± 0.00 | 0.85 ± 0.01 | 0.65 ± 0.06 | 0.64 ± 0.08 | 1.12 ± 0.13 | 0.51 ± 0.03 | 0.008 ± 0.000 |
| Tyr | 2.39 ± 0.03 | 0.25 ± 0.09 | 0.16 ± 0.02 | 0.34 ± 0.08 | 0.64 ± 0.09 | 1.43 ± 0.12 | 0.04 ± 0.06 | 1.7 5 ±0.23 | 1.73 ± 0.52 | 2.16 ± 0.31 |
| ∑NEAA | 24.4 | 8.45 | 12.3 | 14.6 | 51.1 | 42.5 | 42.6 | 33.8 | 58.4 | 75.8 |
| Total Amino acids | 38.9 | 15.8 | 18.3 | 22.2 | 76.0 | 61.8 | 72.7 | 66.8 | 83.6 | 111 |

| Amino acids | *Fucus guiryi* | *Pelvetia canaliculata* | *Halopteris scoparia* | *Gongolaria baccata* | *Cladostephus spongiosus* | *Ericaria selaginoides* |
| --- | --- | --- | --- | --- | --- | --- |
|  |  |  |  |  |  |  |
| His | 0.49 ± 0.04 | 0.02 ± 0.00 | 0.28 ± 0.01 | 0.54 ± 0.06 | 0.24 ± 0.02 | 0.54 ± 0.01 |
| Thr | 0.99 ± 0.02 | 0.84 ± 0.02 | 0.54 ± 0.03 | 0.39 ± 0.01 | 0.27 ± 0.06 | 0.60 ± 0.07 |
| Lys | 2.40 ± 0.06 | 0.84 ± 0.03 | 0.58 ± 0.07 | 1.81 ± 0.03 | 0.76 ± 0.04 | 2.43 ± 0.15 |
| Met | 2.05 ± 0.00 | 0.03 ± 0.01 | 0.35 ± 0.04 | 0.14 ± 0.00 | 0.09 ± 0.02 | 0.21 ± 0.01 |
| Val | 0.69 ± 0.04 | 0.94 ± 0.05 | 0.65 ± 0.06 | 0.55 ± 0.08 | 0.74 ± 0.09 | 0.87 ± 0.13 |
| Ileu | 0.26 ± 0.08 | 0.73 ± 0.03 | 0.54 ± 0.07 | 0.45 ± 0.04 | 0.48 ± 0.02 | 0.50 ± 0.04 |
| Leu | 0.93 ± 0.07 | 1.22 ± 0.16 | 1.11 ± 0.14 | 1.29 ± 0.17 | 0.79 ± 0.15 | 1.40 ± 0.16 |
| Phe | 0.26 ± 0.12 | 0.67 ± 0.11 | 0.22 ± 0.04 | 0.38 ± 0.05 | 6.06 ± 0.69 | 0.87 ± 0.13 |
| ∑EAA | 8.07 | 5.29 | 4.27 | 5.55 | 9.40 | 7.40 |
| Hyptau | nd | 0.01 ± 0.00 | nd | nd | nd | nd |
| Hyp | 0.02 ± 0.00 | 0.01 ± 0.00 | 0.01 ± 0.00 | 0.03 ± 0.00 | 0.01 ± 0.00 | 0.04 ± 0.01 |
| Tau | 0.12 ± 0.00 | 0.07 ± 0.00 | 0.04 ± 0.00 | 0.06 ± 0.00 | 0.05 ± 0.00 | 0.06 ± 0.01 |
| Htau | 0.01 ± 0.00 | 0.02 ± 0.00 | 0.01 ± 0.00 | 0.02 ± 0.00 | 0.01 ± 0.00 | 0.02 ± 0.00 |
| Arg | 0.84 ± 0.15 | 0.74 ± 0.01 | 0.55 ± 0.08 | 0.64 ± 0.01 | 0.38 ± 0.02 | 0.60 ± 0.07 |
| Ser | 0.99 ± 0.04 | 0.90 ± 0.03 | 0.94 ± 0.04 | 0.39 ± 0.02 | 0.70 ± 0.12 | 0.67 ± 0.11 |
| Gly | 3.84 ± 0.17 | 0.96 ± 0.03 | 2.22 ± 0.18 | 0.81 ± 0.12 | 1.76 ± 0.46 | 1.32 ± 0.20 |
| Asp | 3.86 ± 0.58 | 1.46 ± 0.26 | 2.85 ± 0.36 | 3.80 ± 0.25 | 2.00 ± 0.32 | 3.48 ± 0.27 |
| Glu | 4.24 ± 0.36 | 3.74 ± 0.54 | 3.13 ± 0.52 | 2.60 ± 0.12 | 2.80 ± 0.40 | 3.86 ± 0.11 |
| Cys | 0.02 ± 0.00 | 3.63 ± 0.01 | 1.34 ± 0.32 | 1.97 ± 0.13 | 1.81 ± 0.16 | 0.98 ± 0.00 |
| Pro | 1.87 ± 0.41 | 1.34 ± 0.29 | 1.60 ± 0.13 | 1.66 ± 0.30 | 0.76 ± 0.09 | 1.27 ± 0.11 |
| Ala | 2.78 ± 0.17 | 2.57 ± 0.29 | 3.49 ± 0.18 | 3.90 ± 0.35 | 1.49 ± 0.10 | 1.25 ± 0.12 |
| GABA | 0.27 ± 0.02 | 0.09 ± 0.01 | 0.25 ± 0.12 | 0.11 ± 0.10 | 0.11 ± 0.01 | 0.39 ± 0.01 |
| Tyr | 0.55 ± 0.02 | 0.29 ± 0.10 | 0.32 ± 0.01 | 0.15 ± 0.02 | 0.49 ± 0.02 | 0.32 ± 0.09 |
| ∑NEAA | 19.4 | 15.8 | 16.7 | 16.1 | 12.1 | 14.3 |
| Total Amino acids | 27.5 | 21.1 | 21.0 | 22.7 | 21.6 | 21.7 |

**Table S2.** SIRT1 activation / inhibition percentages of the enzymatic protein hydrolysates from algae species.

(Mean of n=3 independent determinations ± std. dev.).

| Alage specie | | % Inhibition | %Activation |
| --- | --- | --- | --- |
| Red Algae | *Porphyra sp* |  | 83.3 ± 1.23 |
|  | *Gigartina pistillata* |  | 68.8 ± 0.98 |
|  | *Chondrus crispus* |  | 56.5 ± 2.32 |
|  | *Mastocarpus stellatus* |  | 32.6 ± 2.47 |
|  | *Palmaria palmata* | 16.0 ± 0.85 |  |
|  | *Gelidium corneum* | 46.9 ± 1.49 |  |
|  | *Plocammium Cartilagineum* | 32.1 ± 2.38 |  |
|  | *Centroceras clavulatum* | 57.7 ± 2.85 |  |
|  | *Halopithys incurva* | 23.4 ± 0.65 |  |
| Median | | **32.1^b^** | **62.7^a^** |
| Green Algae | *Afanizomenon flos-aquae* |  | 62.8 ± 2.85 |
|  | *Caulerpa lentillifera* |  | 98.2 ± 3.44 |
|  | *Codium sp* |  | 88.6 ± 3.87 |
|  | *Dunaliella salina* |  | 63.2 ± 2.14 |
|  | *Spirulina platensis* |  | 66.7 ± 1.45 |
|  | *Chlorella vulgaris* |  | 60.9 ± 0.54 |
|  | *Tetraselmis chui* |  | 39.1 ± 0.85 |
|  | *Auxenochlorella pyrenoidosa* |  | 54.5 ± 1.58 |
|  | *Ulva lactuca* | 41.4 ± 1.14 |  |
|  | *Enteromorpha intestinalis* |  | 60.3 ± 1.45 |
|  | *Codium decorticatum* | 73.5 ± 2.85 |  |
| Median | | **57.5^a^** | **62.8^a^** |
| Brown Algae | *Ascophyllum nodosum* |  | 30.5 ± 2.55 |
|  | *Sargassum fusiforme* |  | 33.6 ± 1.74 |
|  | *Eisenia byciclis* |  | 26.7 ± 0.41 |
|  | *Laminaria ochroleuca* |  | 49.2 ± 0.18 |
|  | *Himanthalia elongata* |  | 41.9 ± 2.22 |
|  | *Undaria pinnatifida* |  | 25.6 ± 2.96 |
|  | *Odonella aurita* |  | 105 ± 3.87 |
|  | *Fucus vesiculosus* | 2.30 ± 0.11 |  |
|  | *Bifurcaria bifurcata* | 80.5 ± 1.85 |  |
|  | *Fucus guiryi* | 46.8 ± 1.37 |  |
|  | *Pelvetia canaliculata* | 55.8 ± 2.49 |  |
|  | *Stypocaulon scoparium* | 63.9 ± 2.97 |  |
|  | *Gongolaria baccata* | 57.7 ± 1.75 |  |
|  | *Cladostephus spongiosum* | 70.2 ± 1.69 |  |
|  | *Cystoseira tamariscifolia* | 34.4 ± 0.65 |  |
|  | *Nanochloropsis sp.* | 4.80 ± 0.17 |  |
| Median | | **55.8^a^** | **37.8^b^** |
